# Genome-wide dynamic nascent transcript profiles reveal that most paused RNA polymerases terminate

**DOI:** 10.1101/2025.03.27.645809

**Published:** 2025-03-28

**Authors:** Rudradeep Mukherjee, Michael J. Guertin

**Affiliations:** a Center for Cell Analysis and Modeling, University of Connecticut Health Center, Farmington, CT, United States of America; b Department of Genetics and Genome Sciences, University of Connecticut Health Center, Farmington, CT, United States of America

**Keywords:** Transcription regulation, Compartment Model, Initiation, Promoter-proximal pausing, Premature termination, PRO-seq

## Abstract

We present a simple model for analyzing and interpreting data from kinetic experiments that measure engaged RNA polymerase occupancy. The framework represents the densities of nascent transcripts within the pause region and the gene body as steady-state values determined by four key transcriptional processes: initiation, pause release, premature termination, and elongation. We validate the model’s predictions using data from experiments that rapidly inhibit initiation and pause release. The model successfully classified factors based on the steps in early transcription that they regulate, confirming TBP and ZNF143 as initiation factors and HSF and GR as pause release factors. We found that most paused polymerases terminate and paused polymerases are short-lived with half lives less than a minute. We make this model available as software to serve as a quantitative tool for determining the kinetic mechanisms of transcriptional regulation.

## Introduction

Gene expression regulation plays a crucial role in determining cell fate during development and influences how cells respond to environmental signals. Early events in the transcription cycle are major determinants of a gene’s transcriptional output. The earliest events of transcription include the formation of a preinitiation complex, promoter opening, rounds of abortive initiation, promoter escape, and ultimately pausing of RNA polymerases 50–100 bases downstream from the transcription start site (1–3). We refer to these early events collectively as *initiation* throughout this study because most molecular measurements in cells cannot distinguish between these transient and unstable states. After initiation, the paused RNA polymerases may either proceed into productive elongation or prematurely terminate and disassociate from the DNA (4–6). Regulation of these steps is crucial for the precise control of transcription.

Previous studies have used imaging or high-throughput genomics assays to investigate and model the kinetics of transcription. Imaging studies track the generation of transcripts linked to fluorescent reporters and use models to interpret the time series of fluorescence signal (7, 8). Others use fluorescence recovery after photobleaching or photoactivatable molecules to determine residence times of RNA polymerases in different stages of transcription (9, 10). Genome-wide nuclear run-on assays, which precisely measure the position and orientation of transcriptionally engaged RNA polymerases, have been combined with inhibitors of initiation and pause release to dissect contributions of each regulated step (11). A recent study employed generative probabilistic modeling of nascent transcription datasets to estimate rates of initiation and pause release (12), highlighting the rate-limiting nature of RNA polymerase escape from the pause region. Despite the numerous studies and diverse methodologies employed, there is no consensus on the absolute and relative rates of these key regulatory steps.

Transcription factors (TFs) interact with various co-factors to coordinate the recruitment and activity of RNA polymerases (13–25). TFs bind to sequence motifs and assemble complexes that facilitate the loading and progression of RNA polymerases (26–29). Understanding the molecular functions of transcription factors, specifically how they contribute to the regulation of RNA polymerase activity at these various steps in early elongation, is crucial to understanding the complex mechanisms that drive gene expression (30, 31). Many foundational studies have leveraged rapidly inducible transcription factors to reveal their regulatory roles by tracking changes in RNA polymerase profiles at target genes (31–36). New chemical and physical methods can more broadly perturb TF activity or degrade TFs, significantly expanding the range of factors whose molecular functions can be studied (37–42). Determining the specific role of each transcription factor and their combinatorial effects on transcription regulation requires methods to acutely activate or perturb TF activity, quantify changes in RNA polymerase activity, and a modeling framework to interpret the data.

**Fig. 1.**
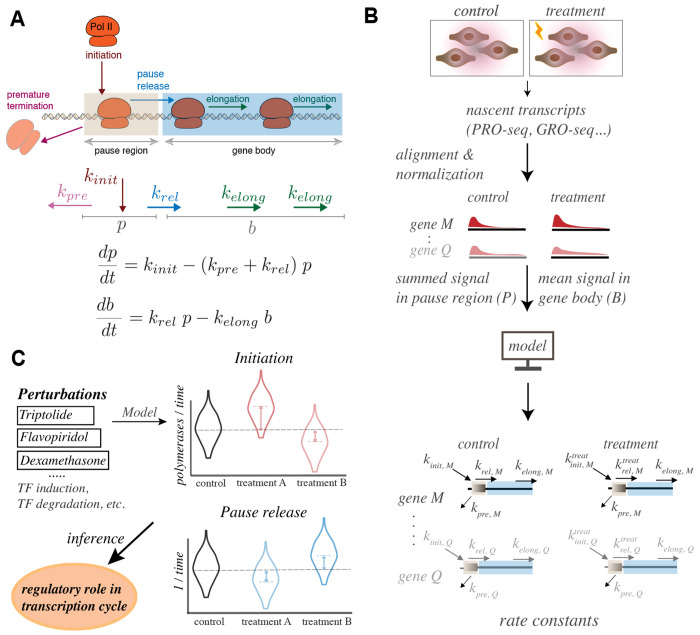
Kinetic modeling of transcription data determines the effect of treatments and perturbations. A) A gene has two compartments in the model–the pause window (*p*) and the gene body (b). RNA polymerase enters the pause region with an initiation rate constant $k_{init}$, prematurely terminates with a rate constant $k_{pre}$, escapes from pause region with a release rate $k_{rel}$, and proceeds through the gene body based on the elongation rate $k_{\text{elong}}$. Herein, we assume that $k_{pre}$ and $k_{\text{elong}}$ remain constant for a gene between conditions. B) The ideal input data for the model is generated from genome-wide nascent transcriptome (PRO-seq) experiments upon acute treatment with a perturbation or stimulus. Standard methods identify differentially expressed genes and the normalized data provides densities of reads in pause and gene body compartments. The model uses these densities to infer initiation and pause release rates for input genes. C) The regulatory effects on pause release and initiation, induced by general perturbations or the acute activation/inhibition of specific transcription factors, are determined by the rate changes explained by the model.

We present a Compartment Model framework, which represents RNA polymerase densities in the pause region and gene body as steady-state values determined by four key transcriptional processes: initiation, pause release, premature termination, and elongation (Fig. 1A). Unless stated otherwise, we define *initiation* as the sequence of events spanning from the incorporation of the first RNA base to the transition into the pause site. For each gene, the model estimates the magnitudes of pause release and initiation rates, as well as changes in these rates for all genes after experimental perturbation (Fig. 1B–C). We describe the framework and validate the model’s predictions through analysis of publicly available experimental data that inhibited either initiation or pause release. Analysis of kinetic triptolide experimental datasets, which inhibit initiation, reveals that premature termination occurs at a faster rate than pause release. We further demonstrate that pause release serves as a critical regulatory step that significantly influences transcription output, but only when the premature termination rate of paused polymerases exceeds the release rate of paused polymerases into the gene body. We analyze publicly available transcription factor activation and perturbation data to confirm that ZNF143 and TBP predominantly regulate initiation, while the glucocorticoid receptor (GR) and *Drosophila* heat shock factor (HSF) regulate pause release. The model is available as a command-line tool that processes relative pause and gene body RNA polymerase densities from two experimental conditions as inputs and provides estimates of initiation rates, pause release rates, premature termination rates, and absolute RNA polymerase densities in pause regions and gene bodies.

## Materials & Methods

### Processing of nascent transcript datasets.

We processed six datasets spanning across a broad range of treatments - triptolide and flavopiridol in mouse v6.5 cells (11), degradation of TBP in HAP1 cells (43) and ZNF143 in HEK293T cells (44), dexamethasone treatment in A549 cells (45) and heat shocked *Drosophila* S2 cells (33). We downloaded the datasets from Gene Expression Omnibus (Table S1) and analyzed the raw data using a general pipeline (46, 47). We normalized signal within datasets using sizeFactors from DESeq2 (48). Since triptolide (12.5 min) and flavopiridol (25 min) treatments cause universal repression, we normalized these datasets by using read counts from gene regions > 50 kb for triptolide (> 100 kb for flavopiridol) as *controlGenes* in DE-Seq2 (48). We chose these gene regions because an elongation rate of 4kb/min is beyond the higher end of contemporary estimates of elongation rates (49). The body length of genes within these data sets were constrained to 25 kb and 50 kb to capture the region where RNA polymerase clears out upon treatment. We provide complete processing steps in the accompanying analysis vignette. We defined Heat Shock Factor (HSF) activated genes in S2 cells (33) by first aligning all data to an *Hsp70* consensus sequence. We mapped the remaining unmapped reads to the dm6 genome, counted reads within genes, and normalized the replicates using DESeq2 sizeFactors (48). We only considered the 249 activated genes selected from Table S2 in Duarte et. al., (33) because these genes were carefully curated for read-through transcription. We considered 65 heat shock activated genes (FDR < 0.001) that are dependent upon the presence of HSF.

### Hsp70 consensus sequence.

We generated the *Hsp70* consensus gene sequence by combining the *Hsp70* sequences from the BDGP6.46 (release 112) reference. We used the start and end coordinates of six Hsp70 genes (*Hsp70Aa/Hsp70Ab, Hsp70Ba, Hsp70Bb, Hsp70Bbb, Hsp70Bc*) and included an additional 50 bases upstream of the reference start site. Using these coordinates, we extracted the six Hsp70 sequences from the reference genome with bedtools getfasta. We then created an alignment file for these sequences using clustalw (50). The consensus sequence was derived by using Biopython’s dumb_consensus() function (51) to the alignment file with a threshold of 0.6.

### Estimation of Pol II densities at pause region and gene body region.

To define the pause region of a gene, we searched for a 50 bp window with the maximum summed PRO-seq signal within a 200 bp region (or 300 bp for GRO-seq) downstream from the most prominent transcription start site (TSS) as defined by primaryTranscriptAnnotation (52). This 50 bp window is designated the *pause region*. The pause density or pause sum is the summed signal across all base pairs in this window. We sum the reads in pause region to accommodate signals from RNA polymerase pausing at any arbitrary position within the pause window. We define the gene body as the region 500 bp from end of the pause window to the inferred transcription termination site (52) or the distance an initiated polymerase can travel during the time of treatment if RNA polymerase elongates at a rate of 2kb/min, whichever is shorter. We define the body density as the mean signal in this window.

### The Compartment Model.

We describe the time evolution of pause and body densities in the following manner:

(1) $$\frac{dp}{dt}=k_{init}-\left(k_{pre}+k_{rel}\right)\cdot p$$

(2) $$\frac{db}{dt}=k_{rel}\cdot p-k_{elong}\cdot b$$

In the above equations, *t* is time, *p* is the density in pause region, *b* is mean density in gene body and $k_{init}$, $k_{rel}$, $k_{pre}$, $k_{elong}$ are rates of initiation, pause release, premature termination, and elongation, respectively. The measurements of pause sum and body densities from sequencing data are assumed to be steady-state values. The steady state assumption is reasonable because the measured rates of these various processes are much faster than the experimental treatment times. At steady-state, we have:

(3) $$\frac{dp}{dt}=0\Rightarrow p=\frac{k_{init}}{k_{pre}+k_{rel}}$$

(4) $$\frac{dp}{dt}=0\Rightarrow b=\frac{k_{rel}}{k_{elong}}\cdot p$$

For each gene, we consider that initiation ($k_{init}$) and pause release ($k_{rel}$) rates are regulated between treatments, while elongation ($k_{elong}$) and premature termination ($k_{pre}$) rates remain constant. Although the range of elongation rates for genes tend to be 1.5-4 kb/min (11, 34, 53–55), we cannot accurately estimate average elongation rates for all genes for each condition. However, we make this assumption of constant elongation rate because RNA polymerase signal within gene bodies correlate well with accumulated RNA-seq signal (56). If elongation rate increased during gene activation, then RNA polymerase density would decrease as mRNA output increases.

Considering two conditions under study as “control” and “treatment”, we have the following relations for change in initiation and pause release rates:

(5) $$fold\;change\left(k_{ini\text{t}}\right)=\frac{k_{init}^{treatment}}{k_{init}^{control}}=\frac{p^{treatment}}{p^{control}}\cdot \frac{k_{\text{pre}}+k_{rel}^{treatment}}{k_{pre}+k_{rel}^{control}}$$

(6) $$fold\;change\left(k_{rel}\right)=\frac{k_{rel}^{treatment}}{k_{rel}^{control}}\;=\frac{b^{treatment}}{b^{control}}\cdot \frac{p^{control}}{p^{treatment}}\;=\frac{\mathrm{fold change}(b)}{\mathrm{fold change}(p)}$$

In Eq. 5, the fraction $\frac{k_{\text{pre}}+k_{rel}^{\text{treatment}}}{k_{\text{pre}}+k_{\text{rel}}^{\text{control}}}$ varies between two values depending upon relative values of premature termination rate ($k_{pre}$) and pause release rate ($k_{rel}$):

(7) $$k_{pre}>>k_{rel}\Rightarrow \frac{k_{pre}+k_{rel}^{treatment}}{k_{pre}+k_{rel}^{control}}\to 1$$

(8) $$k_{pre}<<k_{rel}\Rightarrow \frac{k_{pre}+k_{rel}^{treatment}}{k_{pre}+k_{rel}^{control}}\to \frac{k_{rel}^{treatment}}{k_{rel}^{control}}$$

From Eq. 5–8, fold change in initiation rate ($k_{\text{init}}$) for a gene can vary between fold change in pause density and the fold change in body density:

(9) $$k_{pre}>>k_{rel}\Rightarrow fold\;change\left(k_{init}\right)\to \frac{p^{treatment}}{p^{control}}$$

(10) $$k_{pre}<<k_{rel}\Rightarrow fold\;change\left(k_{\text{init}}\right)\;\to \frac{k_{rel}^{treatment}}{k_{\text{rel}}^{\text{control}}}\cdot fold\;change(p)\;=fold\;change(b)$$

Further, from Eq. 6, the fold change in pause release is an inverse ratio of change in “pausing index” (the ratio of pause density to body density.) As accurate measures of genic elongation rate become available, we can calculate the change in pause release rate by dividing the change in elongation rate by the change in pausing index (Eq. 4). Note that the normalization or scaling of read signals to estimate steady-state *p* and *b* values does not affect the fold change in pause release and bounds on changes in initiation rate. Consequently, the inference of the regulatory effects of a perturbation on initiation and pause release does not depend on the scaling of pause and body densities. For absolute values of rates we derive the following from Eq. 3 & 4:

(11) $$k_{rel}=k_{elong}\cdot \frac{b}{p}$$

(12) $$k_{init}=p\cdot \left(k_{pre}+k_{\text{rel}}\right)\;=p\cdot \left(k_{pre}+k_{elong}\cdot \frac{b}{p}\right)\;=p\cdot k_{pre}+k_{elong}\cdot b$$

From Eq. (11), the absolute value of pause release rate ($k_{rel}$) for a gene depends upon elongation rate (usually between 30-60 bp/sec) and the inverse of the pause to body density ratio. Therefore, the value of pause release rate constant ($k_{rel}$) is also not dependent upon normalization or scaling criteria. From Eq. (12), the initiation rate ($k_{\text{init}}$) depends upon the absolute values of pause and body densities (i.e. the number of RNA polymerase in each compartment). Also, the amount of polymerases releasing into gene body, the effective release rate ($k_{rel}\times p$), provides a way to calculate spacing between polymerases on the gene body in base pairs:

(13) $$\text{Spacing}=\frac{k_{elong}}{k_{rel}\times p}\;=\frac{1}{b}$$

where, elongation rate ($k_{\text{elong}}$) is in units of $\frac{\text{base pairs}}{\text{time}}$ and pause release ($k_{rel}$) has the same time unit. From Eq. 11, the magnitude of occupancy is also equal to reciprocal of body density (*b*).

Further, a relation between premature termination rate ($k_{\text{pre}}$) and pause release rate ($k_{rel}^{\text{control}}$) can be derived from Eq. 5:

(14) $$\frac{k_{pre}}{k_{rel}^{control}}=\frac{fold\;change\left(k_{rel}\right)-X}{X-1}$$

where $X=\frac{\text{fold change}\left(k_{init}\right)}{\text{fold change}(p)}$ with *p* representing the pause density for a gene. Since values and changes in pause release rate can be estimated using elongation rate, pause sums and gene body densities (Eq. 6 & Eq. 11), the rates of premature termination can be assigned to each gene promoter using the relation in Eq. 14. Further, the ratio is not dependent on the scaling criteria used on pause and body densities.

The ratio in Eq. 14 becomes zero or undefined when numerator or denominator becomes zero:

(15) $$X=fold\;change\left(k_{rel}\right)\;=>\frac{fold\;change\left(k_{init}\right)}{fold\;change(p)}=fold\;change\left(k_{rel}\right)\;=>fold\;change\left(k_{init}\right)\;=fold\;change\left(k_{rel}\right)\cdot fold\;change(p)\;=>fold\;change\left(k_{init}\right)=fold\;change(b)$$

While Eq. 15 provides the condition where $\frac{k_{\text{pre}}}{k_{\text{rel}}^{\text{control}}}$ becomes zero, the ratio becomes undefined when denominator in Eq. 14 becomes zero:

(16) $$X=1=>fold\;change\left(k_{init}\right)=fold\;change(p)$$

The values in Eqs. 15–16, which render $\frac{k_{\text{pre}}}{k_{\text{rel}}^{\text{control}}}$ as zero or undefined, represent the extreme bounds on changes in initiation rate as described in Eqs. 9–10. However, transcription is expected to operate within a regime where the rates of premature termination ($k_{pre}$) and pause release ($k_{rel}$) yield a non-zero ratio for $\frac{k_{pre}}{k_{rel}^{control}}.$ Consequently, the extreme cases for the relative values of $k_{pre}$ and $k_{rel}$, as described in Eqs. 9–10, are not considered.

### Pause and body densities as occupancy values.

To infer rates of initiation at different gene promoters we first transformed pause and gene body densities into absolute occupancy values. Occupancy refers to the number of RNA polymerases in the pause region or gene body per gene length. We assume that a fully occupied pause region will contain only one RNA Polymerase and that some genes would approach full occupancy of the pause region. In an ideal dataset without aneuploidy, gene duplications, PCR biases, and sequence mapping ambiguities, the ranked pause sums would saturate to a maximum occupancy. However, over all the datasets examined, the observed ranked pause sums exhibit a sudden uptick at the highest pause densities (Fig. S1A–F). Manual inspection of genes with high pause signals revealed multicopy genes located within non-uniquely mappable regions and genes characterized by highly variable read densities in the gene body. We decided to estimate a fully occupied pause region by fitting ranked pause densities of all expressed genes to a hyperbolic tangent function (y=A⋅tanh⁡(B⋅x)+C) where density of a fully occupied pause region is assumed at saturation = *A*+*C*. We fitted the hyperbolic tangent function to log-transformed ranked pause sums using Scipy’s optimize.curve_fit() function (57).

We reasoned that a saturation value should be robust to amount of input data used to fit the curve, and be minimally influenced by the observed exponential increase in pause sums. For datasets with Unique Molecular Identifiers (UMIs) from which PCR duplicated reads could be confidently removed, the saturation values from incremental fits to pause densities (70th to 100th percentile) showed a modest increase by 1.5- to 4-fold (Fig. S1D–F & Fig. S2A). In contrast, the saturation values from incremental fits for datasets without UMIs varied from 23-fold to 600-fold (Fig. S1A–C & Fig. S2A). The saturation fits for datasets with UMIs tended to stabilize and reach 90th percentile of the ranked paused sums (Fig. S1D–F). Since the saturation values for datasets with UMIs were robust, we used the saturation values obtained from fits to all genes as the maximum occupancy signal. To remain consistent and mitigate the influence of the exponential increase in saturation values, we considered the pause sum at 90th percentile as maximum occupancy signal in datasets without UMIs. Using the maximum occupancy value, we scaled all pause and gene body densities from 0 to 1 to estimate initiation rates (Eq. 12). The assumption that the pause sum saturates at the 90th percentile of ranked pause sums in datasets without UMIs may not be accurate, as the pause sum at the 90th percentile is lower than the pause signals observed for heat shock activated genes (such as *Hsp70*) in *Drosophila* (Fig. S2B&C). Although our initiation rate calculations are based on these assumptions, more accurate estimates can be obtained by calibrating saturation values using datasets with known RNA polymerase densities in specific genomic regions.

### Vignette and Model Availability.

A Python package is currently installable from PyPi: https://pypi.org/project/compartmentModel using the command: python3 -m pip install compartmentModel.

The details of installation, running the model and processing of sequencing data is available this vignette: https://github.com/guertinlab/compartment_model/tree/master/compModel_vignette/compModel_vignette.

The source files for building the package, figures, and UCSC Genome Browser track hubs are available: https://github.com/guertinlab/compartment_model. The analysis vignette is available here: https://guertinlab.github.io/compartment_model/compModel_vignette/compModel_vignette/compartmentModel_vignette.html

## Results

### Direct inhibition of pause release and initiation verifies the model predictions.

**Fig. 2.**
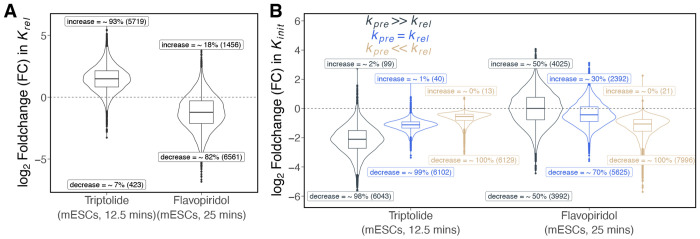
Inhibition of initiation and pause release validate the model. A) Flavopiridol treatment decreases pause release for ~82% of repressed genes, consistent with its known inhibitory effect on CDK9. Inspection of genes with increased pause release reveal a decrease in pause density for all genes (Fig. S3A–C). This unexpected reduction in pause density is attributed to spiky signals in the pause region or insufficient reads to accurately define the pause window (Fig. S3D–I). Triptolide increases pause release at most genes. Although this was not expected, this phenomenon could be a result of a general compensatory mechanism to increase transcription when initiation is inhibited. B) The bounds on changes in initiation rate are estimated with distinct relative values of pause release ($k_{\text{rel}}$) and premature termination rate ($k_{\text{pre}}$) (Materials & Methods). As expected, nearly all repressed genes (>99%) decrease their initiation rate upon triptolide treatment. Flavopiridol does not show any clear effect on initiation if $k_{\text{pre}}\gg k_{rel}$, but appears inhibitory if $k_{\text{pre}}\ll k_{rel}$ or $k_{\text{pre}}=k_{\text{rel}}$.

The compartment model we developed interprets the mechanistic steps of RNA polymerase initiation, pause release, premature termination, and elongation as rate constants that explain observed changes in nascent transcription after acute perturbation and treatments. To validate the model predictions for changes in pause release rates (Eq. 6), we analyzed GRO-seq data following the inhibition of pause release with flavopiridol (11). Flavopiridol is a kinase inhibitor that targets CDK9 (58, 59), a key component of the positive transcription elongation factor b (P-TEFb) complex. P-TEFb promotes the release of paused RNA polymerase II into productive elongation, partially through the phosphorylation of the RPB1 C-terminal domain (4, 58, 60). Treatment with flavopiridol (0.3 μM) for 25 minutes results in a decrease of the pause release rate for ~82% of repressed genes, with a median rate decrease of ~57% (Fig. 2A, Fig. S3, & Fig. S4A). Additionally, we observed a modest reduction in the initiation rate, with a median decrease ranging from 0% to 52% for 50-100% of repressed genes, depending on the relative rates of premature termination and pause release (Fig. 2B).

The model links changes in a gene’s initiation rate to changes in body and pause densities. The change in initiation rate for a gene is constrained by the fold change in either pause density or body density, depending on the relative rates of premature termination and pause release (Eq. 9 and Eq. 10). To validate the model predictions, we analyzed genome-wide nascent transcriptome data after acute treatments with an inhibitor of initiation, triptolide (TRP)(11). Triptolide blocks initiation by inhibiting XPB helicase (63), a component of TFIIH (64, 65). Treatment with 0.5 μM triptolide for 12.5 mins (11) causes the expected decrease in initiation rate for more than 98% of repressed genes, with a median decrease of ~77% when premature termination is faster than pause release ($k_{pre}\gg k_{rel}$, Fig. 2B, Fig. S4B). When pause release is comparable to or faster than premature termination ($k_{rel}=k_{pre}$ or $k_{rel}\gg k_{pre}$), triptolide treatment leads to a median decrease in initiation rate of approximately 54% or 32%, respectively (Fig. 2B). Since initiation rate changes are constrained within a range and are dependent on relative rates of premature termination and pause release, we examined the results more closely to better understand these relative rates.

### Inhibition by triptolide indicates that premature termination is faster than pause release.

**Fig. 3.**
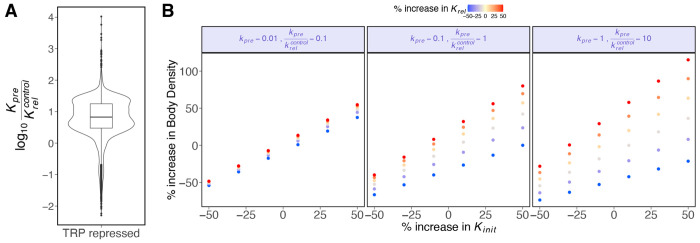
Experimental data and the model equations indicate that premature termination is faster than pause release. A) We set all TRP-repressed genes to have fold change (FC) in initiation rate ($k_{\text{init}}$) close to 0.25 (61). We calculated ratio of premature termination and pause release rates (Eq. 14). The median value of $\frac{k_{pre}}{k_{rel}^{control}}$ was 6.7 with an inter-decile range of 1.2 - 30. B) Changes in pause release affect changes in body density (transcription output) when premature termination *$k_{pre}$* is faster ($k_{pre}\gg k_{rel}$). When $k_{pre}$ is relatively slower than *$k_{rel}$,* change in body density is closely dependent upon changes in *$k_{\text{init}}$* and is minimally affected by changes in $k_{rel}$. As termination *$k_{pre}$* becomes faster than pause release *$k_{rel}$*, the body density becomes dependent on changes in *$k_{rel}$* and approaches the product of changes in initiation *$k_{\text{init}}$* and pause release *$k_{rel}$* rates. Pausing is regulated to control expression output in metazoans (62), so these results support premature termination being faster than pause release at most genes.

Since changes in initiation rate are defined by relative values of premature termination and pause release (Eqs. 9–10), we sought to determine which rate is likely to be faster. To quantify the relationship between $k_{pre}$ and $k_{rel}$, we considered changes in the initiation rate (Eq. 5) for repressed genes after triptolide treatment (Fig. 2B). Specifically, we reasoned that the fold change in initiation rate for all repressed genes will be close to 0.25, based on the results that initiation is decreased by ~75% after 10 minutes of 1μM triptolide treatment (61). Approximately 55% (3367 of 6142) of repressed genes had fold-change in initiation rate bounds spanning 0.25; 45% (2775 of 6142) of the genes had bounds that didn’t include 0.25 (Fig. S5). For genes with bounds of changes in initiation rate spanning 0.25, we assumed their change in initiation rate after triptolide treatment to be 0.25. For genes with bounds on initiation rate changes not spanning 0.25, we set their changes in initiation rate to the bound closest to 0.25, with a 5% offset (i.e; $\mathrm{fold change}(k_{\text{init}})=(1\pm 0.05)\times \mathrm{closestBound}$). The 5% buffer prevents $\frac{k_{\text{pre}}}{k_{\text{rel}}^{\text{control}}}$ values of zero or undefined as described in Eqs. 15–16. We then calculated $\frac{k_{\text{pre}}}{k_{\text{rel}}^{\text{control}}}$ (Eq. 14) which resulted in a median value of 6.7, an inter-decile range of 1.2 - 30, and $k_{pre}>k_{rel}$ for 90% of genes (5509 of 6142) (Fig. 3A). This trend is consistent among genes that are classified according to their expression level (Fig. S6). This suggests that the rate of premature termination is much faster than pause release at the majority of gene promoters, and on average only one out of seven paused polymerases proceeds into productive elongation. These results are consistent with previous reports that suggest premature termination rates are 4-12 times faster than pause release rates (9, 66) (Table S2).

The result of flavopiridol inhibition on pause release also suggests that $k_{pre}\gg k_{rel}$. The pause inhibition data was generated with 300 nM flavopiridol (FP) for 25 minutes, which should cause a decrease in pause release without preferentially stimulating or inhibiting initiation. However, our analysis indicates that if $k_{pre}\ll k_{rel},$ then nearly every gene would have to decrease initiation after FP treatment to explain the observed changes in pause and gene body densities (Fig. 2B) (11). While flavopiridol may interact with other kinases, such as CDK7 (59), at this concentration flavopiridol is unlikely to cause a decrease in initiation at nearly all genes. Moreover, the observed increase in RNA polymerase density at pause sites following flavopiridol treatment (11) contrasts with the decrease in pause density reported for CDK7 inhibition (67). Both the triptolide and flavopiridol analyses support the conclusion that premature termination occurs at a rate substantially faster than pause release.

### Change in pause release affects transcription output when premature termination is faster than pause release.

To better understand how the rates of initiation, pause release, and premature termination collectively influence gene expression, we examined how changes in these rates determine transcription output. We use RNA polymerase density in the gene body as a proxy for change in transcription output because transcript levels are determined by the product of three quantities: (a) the fold change in initiation rate ($k_{init}$), (b) the fold change in pause release rate ($k_{rel}$) and (c) the inverse of fold change in “net exit rate” from the pause region, $\frac{k_{\text{pre}}+k_{rl}^{\text{control}}}{k_{\text{pre}}+k_{rel}^{\text{treatment}}}$. Depending on the relative values of pause release $\left(k_{rel}\right)$ and termination rate $\left(k_{\text{pre}}\right)$, the inverse of fold change in exit rate $\left(\frac{k_{pre}+k_{rel}^{\text{control}}}{k_{\text{pre}}+k_{rel}^{\text{treatment}}}\right)$ is either $1(k_{\text{pre}}\gg k_{rel})$ or the inverse of fold change in pause release $(\frac{k_{\text{rel}}^{\text{control}}}{k_{\text{rel}}^{\text{treatment}}}$ when $k_{pre}\ll k_{rel})$ (Eq. 7–8)

Thus, when pause release is much faster compared to termination ($k_{rel}\gg k_{\text{pre}}$), the change in transcription output closely mirrors the change in initiation rate and variations in pause release ($k_{rel}$) have minimal impact on transcription output (Fig. 3B left panel). As $k_{\text{pre}}$ becomes faster than $k_{rel}$, changes in pause release ($k_{rel}$) become more influential, and transcription output approaches the product of change in initiation and change in pause release rate (Fig. 3B right panel). These results demonstrate that regulating pause release has a bigger effect on gene expression output when premature termination occurs at a relatively faster rate.

These analyses, taken together with the analysis of pause-inhibition and initiation-inhibition data, as well as previous work (Table S2), suggests that premature termination is faster than pause release at most genes. Therefore, we maintain this assumption throughout the main figures and text, meaning that changes in initiation rate are interpreted as fold change in pause densities as measured by PRO-seq (Eq. 9).

### Mechanistic functions of transcription factors.

**Fig. 4.**
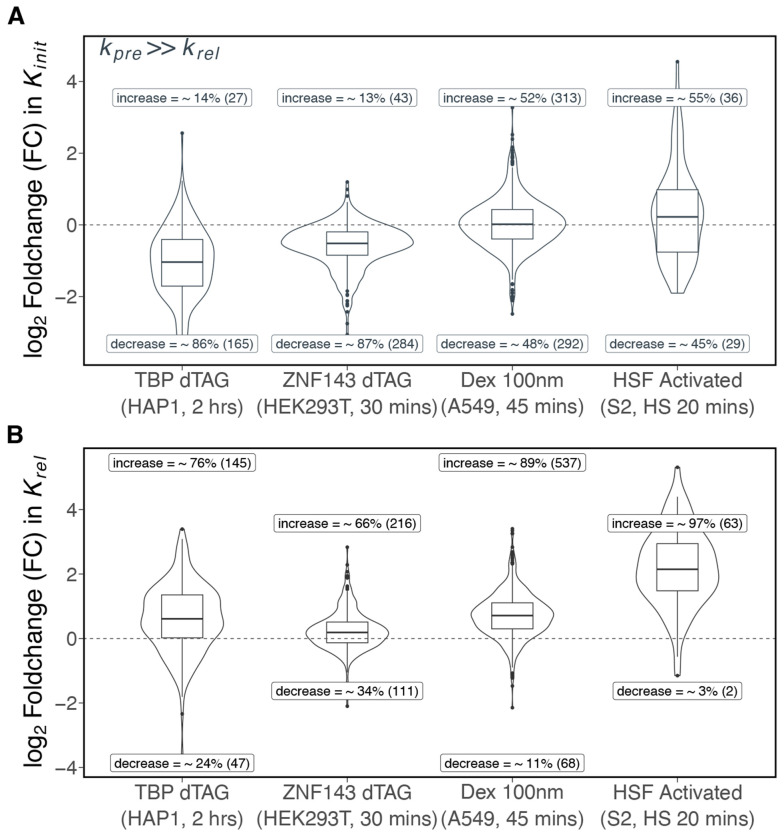
Modeling kinetic PRO-seq data determines the regulatory roles of activated and degraded transcription factors. A) Degradation of ZNF143 (44) and TBP (43) reduces initiation for approximately 90% of repressed genes in each dataset. Dexamethasone-activated genes (45) and Heat Shock Factor (HSF)-activated genes in S2 cells (33) do not show a clear direction of change in initiation rate, suggesting that *Drosophila* HSF and human glucocorticoid receptor (GR) may not regulate initiation. Fig. S8 contains the estimated fold change in initiation rates if premature termination is less than pause release. B) Dexamethasone (45) enhances pause release in approximately 89% of activated genes, indicating that GR’s role is to regulate pause release. In *Drosophila* S2 cells, HSF activates target genes by facilitating pause release. Degradation of TBP and ZNF143 tends to increase pause release for repressed genes, similar to the effect seen in repressed genes after triptolide treatment (Fig. 2.)

To determine the regulatory roles of transcription factors, we analyzed existing PRO-seq datasets that rapidly degraded TBP and ZNF143 (43, 44). The degradation of TBP reduces the initiation rates for more than ~86% of repressed genes (Fig. 4A, Fig. S7A), highlighting TBP’s established role in initiation (68). Similarly, the degradation of ZNF143 led to a decrease in initiation rates for over ~87% of repressed genes (Fig. 4A, Fig. S7B), indicating that ZNF143 stimulates initiation. Previous work proposed the role of ZNF143 as an initiation factor by demonstrating that ZNF143 influences transcription start site (TSS) selection, with alternative TSSs being utilized in repressed genes following ZNF143 degradation (44).

Next, we investigated the function of glucocorticoid receptor (GR) by analyzing PRO-seq data after acute dexamethasone (Dex) treatment (45). Upon binding dexamethasone, GR translocates to the nucleus, binds DNA and directly activates transcription (69–71). After 45 mins of dexamethasone treatment in A549 cells (45), pause release rate increased for 89% of activated genes (Fig. 4B, Fig. S7C) with no clear change in initiation rate for Dex-activated genes (Fig. 4A), indicating that GR predominantly activates its target genes by stimulating productive release of paused RNA polymerases.

Promoter-proximal pausing was first characterized at *Drosophila* heat shock genes (2) and the *Drosophila* heat shock factor (HSF) is known to stimulate pause release (33, 72, 73). Our analyses confirms that nearly all (~97%) HSF-activated genes in *Drosophila* S2 cells show an increase in pause release upon heat shock (Fig. 4B, Fig. S7D). Taken together, these analyses of existing kinetic PRO-seq data demonstrates the utility of this approach when assigning molecular functions to transcription factors.

### Inferring absolute and effective pause release rates.

**Fig. 5.**
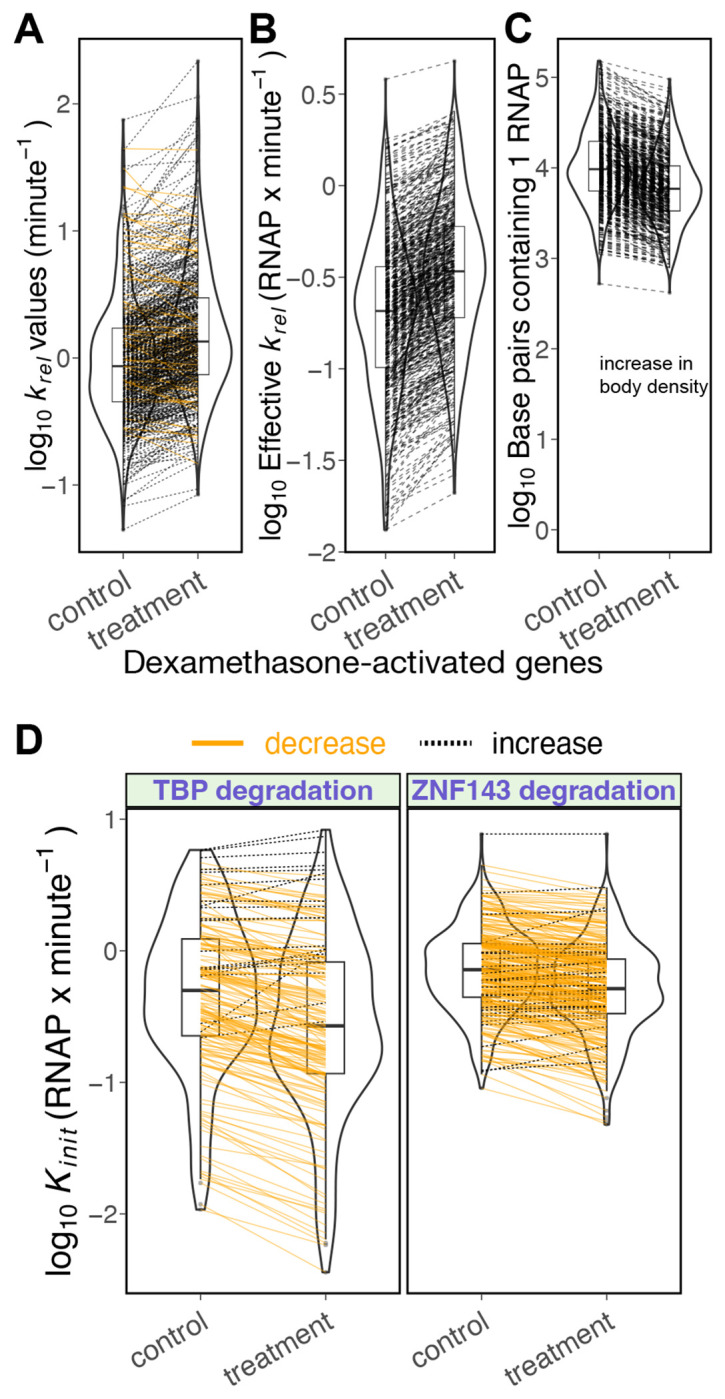
Rates of initiation and pause release can be estimated using steady-state densities of pause and gene body regions. A) Assuming an elongation rate of *$k_{\text{elong}}=2000bp/min$*, the median pause release rates of Dex-activated genes increase from 0.86 events/min (control) to 1.35 events/min. 89% of the Dex-activated genes (537 out of 605) increase their pause release rate. B) The effective pause release rate represents the release of RNA polymerase into the gene body and is calculated as the product of the pause release rate constant and the scaled pause density. The pause and body densities was transformed into occupancy levels of RNA polymerase (Fig. S1E). The median effective pause release rate for Dex-activated genes increases 1.6-fold, from 0.21 RNAP/min to 0.34 RNAP/min under treatment. All Dex-activated genes increase their effective pause release rate. C) The density in gene body increased after dexamethasone treatment with median density increasing from 1 RNAP every 9.6kb to 1 RNAP every 5.8kb. The inter-decile ranges were 1 RNAP every 3.1 kb - 36.3 kb in untreated conditions, increasing to 1 RNAP every 1.8 kb - 20.3 kb after dexamethasone treatment. D) Degradation of TBP in HAP1 cells and ZNF143 in HEK293T cells decreases the median initiation rate from 0.5 RNAP/min to 0.27 RNAP/min and 0.71 RNAP/min to 0.51 RNAP/min respectively.

Our model estimates the absolute values of pause release rates as the product of elongation rates and the inverse of a gene’s *pausing index* (Eq. 11). Since these estimates are derived from the ratio of body to pause densities, they are independent of the scaling or normalization criteria applied to genomic data. We considered 2000 bp/min a representative elongation rate and we kept this constant for all genes because we cannot estimate accurate gene-specific elongation rates. We report the pause release rates before and after Dex treatment because GR regulates pause release (Fig. 4B) (45). The median pause release rate constants increase from 0.86 events/min to 1.35 events/min (Fig. 5A), with an interdecile range of 0.27 - 4.1 events/min in untreated conditions and 0.42 - 7 events/min after dexamethasone treatment.

We also estimated an effective pause release rate, $k_{rel}\times P$, representing the number of polymerases entering productive elongation at steady state. Since calculating effective pause release requires an estimation of pause densities as absolute occupancy values, we used a saturation fit to scale pause densities (Fig. S1E) (Materials & Methods). Upon dexamethasone treatment, the median effective pause release rate of Dex-activated genes increased 1.6-fold from 0.21 RNA polymerase (RNAP) /min to 0.34 RNAP/min (Fig. 5B) with an inter-decile range of 0.06 - 0.63 RNAP/min under untreated conditions and 0.09 - 1 RNAP/min after dexamethasone treatment. Effective pause release rates increase for all Dex-activated genes (Fig. 5B). Ninety percent of Dex-activated genes have ≤ 1 RNAP entering the gene body per minute (Fig. 5B).

Next, we calculated RNA polymerase occupancy levels in the gene body of Dex-activated genes in both conditions (Eq. 13). Based on the effective pause release rates, the median estimated occupancy in untreated cells was 1 RNA polymerase per 9.6 kilobases (kb), with an interdecile range of 1 RNA polymerase per 3.2 kb - 33.3 kb (Fig. 5C). The density of RNA polymerase in the gene body increased after dexamethasone treatment to median of 1 RNA polymerase every 5.8 kb and inter-decile range of 1 RNA polymerase every 1.8 kb - 20.3 kb (Fig. 5C). These results align with previous imaging studies showing that two-thirds of nascent RNA transcripts are spaced more than 12 kb apart from another RNA polymerase, while the remaining 33% of transcripts exhibit densities ranging from 1 RNA polymerase per kb to 1 RNA polymerase per 8 kb (74, 75). Previously reported “initiation” rates of 0.16–0.4 events/min in single-molecule imaging studies (76) can be interpreted as effective pause release rates, corresponding to a body density of 1 RNA polymerase every 5–12.5 kb. Another study estimated effective pause release rates of 0.19–0.29 events/min, yielding a body density of 1 RNA polymerase every 6.9–10.5 kb (77). Our calculated pause release rates and genomic estimates of RNA polymerase occupancy align with independent imaging and single-molecule studies, underscoring the validity of our approach in converting unitless genomic RNA polymerase profiling values to absolute RNA polymerase occupancy estimates.

### Inferring absolute initiation rates.

The initiation rate estimate incorporates premature termination rates, elongation rates, with pause and body densities as occupancy values (Eq. 12) (Materials & Methods). We concluded faster premature termination rates (*k_pre_*) than pause release rates (*k_rel_*) at gene promoters using experimental data from triptolide-treated cells (Fig. 3A). For the rapid degradation of TBP and ZNF143 datasets, we assumed that premature termination is 6.7x pause release (Fig. 3A). Upon TBP degradation in HAP1 cells, the median initiation rate decreased from 0.5 events/min to 0.27 RNAP/min with inter-decile ranges of 0.07 - 2.7 RNAP/min under untreated conditions and 0.04 - 2 RNAP/min upon TBP degradation (Fig. 5D). While, upon ZNF143 degradation in HEK293T cells, the median initiation rate decreased from 0.71 RNAP/min to 0.51 RNAP/min with inter-decile ranges of 0.3 - 1.9 RNAP/min under untreated conditions and 0.17 - 1.4 RNAP/min upon ZNF143 degradation (Fig. 5D). These calculations indicate that initiation rates are, on average, half of pause release rate constants, but the distributions of these rates largely fall within comparable ranges.

### Most paused polymerases turn over in less than a minute.

**Fig. 6.**
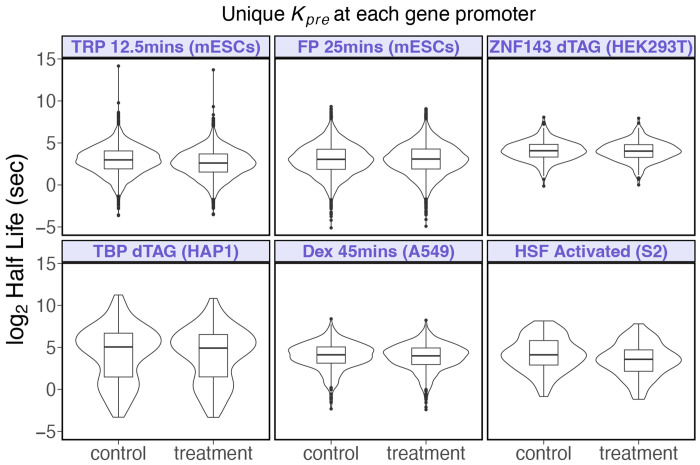
Most paused polymerases turn over rapidly. Assuming premature termination rates at each gene promoter as 6.7 times faster than pause release rates (Fig. 3A), the median half-life of paused polymerases across all datasets and treatments is 8.2 seconds, with an inter-decile range of 1.6 to 36.2 seconds. For repressed genes under triptolide treatment, the median half-life decreased from 7.9 seconds to 6.1 seconds. For HSF-activated genes, the median half-life decreased from 17.4 seconds to 12 seconds. The half-lives in other datasets showed only minor changes after treatment.

The residency time of a paused RNA polymerase is determined by the rates of premature termination and pause release. Based on Fig. 3A, we considered $k_{pre}=6.7\times k_{rel}$ at each gene promoter to calculate the residency time of paused RNA polmerase. The median half-lives of paused RNA polymerases, calculated as ${log}_{2}/\left(k_{pre}+k_{rel}\right)$, across all treatments was approximately 8.2 seconds (Fig. 6), with an inter-decile range of 1.6 to 36.2 seconds, indicating that most paused polymerases turn over within a minute. Premature termination rates primarily determine half-lives rather than pause release rates. For instance, at *$k_{\text{pre}}=10\times k_{rel}$* (Fig. S9), the turnover time and interdecile range decrease by an approximate factor of 1.5 (i.e., 10/6.7). Although several studies are consistent with our estimates of short lived paused polymerases (9, 61, 78), others report longer half-lives (10, 11, 66, 79). In the discussion we provide possible explanations for the disparate estimates in an attempt to reconcile seemingly inconsistent findings.

### Parameter sensitivity analysis supports the robustness of our conclusions.

The three values that we base many of these calculations and our conclusions upon are: 1) the average elongation rate of a gene; 2) the efficacy of triptolide inhibition; and 3) the absolute density of RNA polymerases in the pause region. We varied these values over a wide range to determine how changing these parameters influence rates, ratios of rates, and our general conclusions.

In the previous sections we assumed that the average elongation rate is 2kb/min. However, elongation rates can vary between genes and within a gene (34, 49, 80, 81). Our analysis ignores the fact that elongation rate varies within a gene because we average the gene body density, which effectively averages the elongation rate in our model. We varied the average elongation rate from 1-5kb/min to determine how elongation speed influences other rates and our conclusions. In short, changing the elongation rate causes linear and proportional changes in all other rates (Fig. S10), since pause release is linearly dependent on elongation (Eq. 11) and premature termination is related to pause release (Eq. 14). For example, if we increase elongation rate by 2 fold, then all other rates will change by two-fold. The values of elongation rate have no effect on $k_{\text{pre}}/k_{rel}$ (Eq. 14), fold changes in initiation (Eq. 9), or fold change in pause release (Eq. 6) between experimental conditions. However, faster elongation rates would correspond to shorter residency times of paused RNA polymerase due to faster termination and pause release (Fig. S10A&B).

We assumed that 12.5 minutes of 0.5 μM triptolide caused an average fold-change in the initiation rate of 0.25 for triptolide-repressed genes in mESC cells (11). This estimate is based on an *in vitro* estimate of genome-wide decrease in initiation after a time course (1, 3, 10 and 30 mins) of 1μM triptolide treatment (61). Uncertainty in this parameter will affect our estimates of absolute premature termination rates, absolute initiation rates, and the premature termination to pause release ratio (Fig. S11). If triptolide treatment is less effective than we assume (i.e. fold-change in initiation is >0.25), then the premature termination rate decreases (Fig. S11A, Eq. 14). The pause release rate is only dependent upon elongation rate, so this remains unchanged. If triptolide causes a modest 0.45 fold-change in initiation, then approximately half of genes have a *$k_{pre}>k_{rel}$* (i.e. termination and pause release rates are comparable) (Fig. S11A). The reciprocal is also true: if we are underestimating triptolide efficacy, then premature termination of a paused RNA polymerase is more likely than we report. Our estimate of triptolide efficacy is based on experimental data and 75% inhibition spans the empirically determined bounds of initiation rate fold change (Fig. S5), but other studies assume that this same triptolide treatment is nearly complete and more rapid (11). Although we only focus on triptolide-repressed genes herein, all genes are likely differentially affected by triptolide treatment, some completely inhibited and others insensitive. In the future, gene-specific measurements or estimates are needed to quantify the relationship between termination and pause release at individual genes.

We used a saturation function to estimate “full” occupancy of the pause region, which allowed us to calculate the absolute density of RNA polymerase in the gene body (Fig. S1A–F). Since the triptolide dataset (11) doesn’t have UMIs, we had considered 90th percentile as saturation to be consistent with saturation values observed in datasets with UMIs (Fig. S1D–F). To investigate how saturation values may affect occupancy, we varied them by 5-fold in each direction (Fig. S12). Based on pause signal of *x* arbitrary units at the 90th percentile, we considered pause sums at 48, 70, 90, 95, 99.8 percentiles of ranked pause sums (P = 0.2*x*, 0.4*x*, *x*, 1.8*x* and 5*x*). The ratio of premature termination rate to pause release rate remains stable over this wide range of absolute RNAP density estimates (Fig. S12A), with only modest <2-fold differences in both rates over the 25-fold range (0.2*x* to 5*x*) (Fig. S12B&C). Initiation rate is more sensitive to the RNAP density, decreasing proportionally as RNA polymerase density estimates increase (Fig. S12D).

Scaling absolute pause occupancy proportionally affects gene body density calculations. By inferring the RNAP density in the pause region, we can calculate the median values of RNA Polymerase density within genes. These median gene body densities equate to 1 RNAP every 1.5 kb (48 percentile; 0.2*x*), 1 RNAP every 5.9 kb (90 percentile; *x*), and 1 RNAP every 28.3 kb (99.8 percentile; 5*x*) (Fig. S12E). The 90th percentile saturation values (Fig. 5C, Fig. S12E) equate to gene body densities that are supported by multiple imaging studies: 1 RNAP every 8-12 kb (74), 1 RNAP every 5-12.5 kb (76) and 1 RNAP every 6.9-10.5 kb (77). Although, absolute RNAP occupancy estimates influence all calculations except the *$k_{pre}/k_{rel}$* ratio, defining “full” occupancy using the 90th percentile of pause density–guided by saturation curve estimates (Fig. S1)–yields gene body densities that align best with independent RNA polymerase occupancy measurements.

## Discussion

**Fig. 7.**
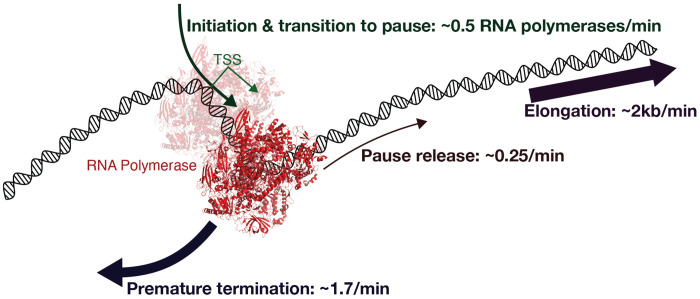
A kinetic model of initiation, termination, and early elongation. The cartoon represents the conclusions from our modeling of kinetic nascent transcriptome data. The initiation rate of 0.5 RNA polymerases per minute assumes a non-limiting amount of RNA polymerase available to initiate. The translucent RNA polymerase represents pre-initiation or abortive initiation complexes; so the term *initiation* rate within our model refers to the series of events that have to occur for an RNA polymerase to successfully proceed into the pause region. The average pause release rate constant is 0.25/minute and the average termination rate of pause RNA polymerases is approximate 7 time faster at 1.7/minute. These rates are derived from well established rates of elongation (~2 kilobases/minute) averaged over a gene body, experimental triptolide efficacy data, and empirical estimates of fully occupied pause sites.

The results of this study provide insights into the kinetic mechanisms of transcriptional regulation, with a focus on the fate of paused RNA polymerases. Our modeling framework, validated with kinetic PRO-seq datasets, reveals that most paused polymerases terminate prematurely rather than progressing into productive elongation (Fig. 7). We found that the rate of premature termination ($k_{pre}$) is significantly faster than the rate of pause release ($k_{rel}$) for most genes. By leveraging triptolide inhibition data, we estimated the ratio of premature termination to pause release rates ($k_{\text{pre}}/k_{rel}$) and found that premature termination occurs approximately seven times more frequently than productive elongation. This aligns with imaging-based studies reporting rapid turnover of paused polymerases within seconds (9). Moreover, the dependency of transcriptional output on pause release rate changes further supports the predominance of premature termination. The relative rates of termination versus pause release has significant implications for understanding transcriptional control. Transcription factors that enhance pause release, such as HSF and GR, have greater influence on gene expression in contexts with fast termination rates. The ability of our model to quantify these rates provides a framework for determining how different factors modulate transcription.

Our framework was validated using datasets in which initiation and pause release were directly inhibited. The effects of triptolide on initiation and flavopiridol on pause release were consistent with known molecular mechanisms, demonstrating that our model accurately captures kinetic changes in transcription. Furthermore, the model successfully classified transcription factors based on their regulatory roles, confirming TBP and ZNF143 as initiation factors and HSF and GR as regulators of pause release.

### Model limitations.

Sensitivity analyses further support the robustness of our conclusions. While absolute values of rates depend on estimates of elongation speed and triptolide efficacy, the key finding that termination is faster than pause release remains consistent across a broad range of estimates. Moreover, our calculation of gene body RNA polymerase densities from the modeling align with previous imaging studies, reinforcing the validity of our RNA polymerase occupancy estimates.

One limitation of our current framework is that it does not explicitly take transcriptional bursting into account. Transcription occurs in stochastic bursts, where genes switch between active and inactive states, leading to fluctuations in RNA polymerase occupancy (82). Although our model assumes steady-state transcription rates, it can be adapted to incorporate bursting kinetics if the burst frequency and duration are known for a given gene. For example, if 20% of a gene’s alleles are active, then the saturation curve would be re-calibrated so that complete occupancy would be considered 20% of the estimated full occupancy. Future work could integrate live cell imaging data to measure these values and refine the model to more fully capture dynamic transcriptional regulation for each gene.

### Reconciling disparate estimates of rates and pause residency times from previous studies.

We found that premature termination is much faster than pause release (Fig. 3A) and paused RNA polymerases turn over rapidly, on the order of tens of seconds (Fig. 6). Some previous studies report slower pause release and premature termination rate constants (Table S2) (66), with estimated half-lives in the range of several minutes (66, 79). A key distinction between these studies and our approach is that they exclusively measured capped short nascent RNA (66, 79), while we quantify all engaged RNA polymerases that are competent to elongate (83, 84). We speculate that paused RNA polymerases with capped RNA are less likely to prematurely terminate than polymerases with uncapped RNA, potentially accounting for the observed differences. If nearly all initiated polymerases terminate prematurely (9) (Fig. S13), the slow turnover rates observed in (66, 79) may primarily reflect the termination dynamics of the minority population of paused RNA polymerases with capped RNA, with minimal contribution of pause release to measurements in both untreated and flavopiridol conditions.

Another study that reported longer half-lives in minutes had used 0.5μM triptolide treatment over several time points (11). Since the initial publication of this study, comparable times and concentrations of triptolide were shown to be insufficient to fully inhibit initiation (61). Our analysis also provides an upper bound for initiation inhibition, which supports these *in vitro* measurements of triptolide efficacy (61). Anything less than immediate and complete inhibition of initiation would cause an inflation of pause residency time because all newly initiated RNA polymerases will be considered stably paused.

A live cell imaging study used a photoactivatable GFP-RNA Pol II to measure pause stability at an uninduced and heat shock-induced *Hsp70* transgene (10). This work was performed in polytene chromosomes, averaging the RNAPII signal over a thousand copies of *Hsp70*. This analysis assumes that paGFP-RNAPII will dissociate from the locus upon termination, but if terminated RNAPII reinitiates at a proximal *Hsp70* gene, it will not contribute to the decay of GFP signal from the locus. This will result in an inflated half-life of paused polymerases because any local reinitiation will be considered stably paused. They also used a biochemical fractionation experiment upon inhibition of initiation with 10μM triptolide to quantify decay of nascent paused RNA (10). Again, subsequent studies found that even 10μM triptolide is not sufficient to immediately and completely inhibit initiation (61). This leads to a deflated decay rate because newly initiated RNA polymerases are indistinguishable from polymerases that are stably paused.

### Future Directions.

Although our model is based on known mechanisms of initiation and early elongation and provides valuable insights into transcription dynamics, future work could refine these estimates using direct measurements of elongation rates at individual genes. Single-molecule imaging approaches could complement our genomic estimates by providing real-time observations of polymerase behavior. Expanding this model to incorporate gene-specific variations in elongation and bursting kinetics would enhance its predictive power.

By quantifying initiation, pause release, and termination rates, our framework and corresponding compartmentModel software are powerful tools for dissecting the kinetic mechanisms of transcription and determining the molecular functions of transcription factors.

## Supplementary Material

1
