# Supplementary material for: Genome-wide dynamic nascent transcript profiles reveal that most paused RNA polymerases terminate
Source: bioRxiv. 2025 Mar 28:2025.03.27.645809. Preprint. [Version 1] doi: 10.1101/2025.03.27.645809 (PMC11974822; doi:10.1101/2025.03.27.645809)
Supplement: 1 [file NIHPP2025.03.27.645809v1-supplement-1.pdf]

bioRxiv preprint doi: https://doi.org/10.1101/2025.03.27.645809; this version posted March 28, 2025. The copyright holder for this preprint (which was not certified by peer review) is the author/funder, who has granted bioRxiv a license to display the preprint in perpetuity. It is made available under aCC-BY 4.0 International license.

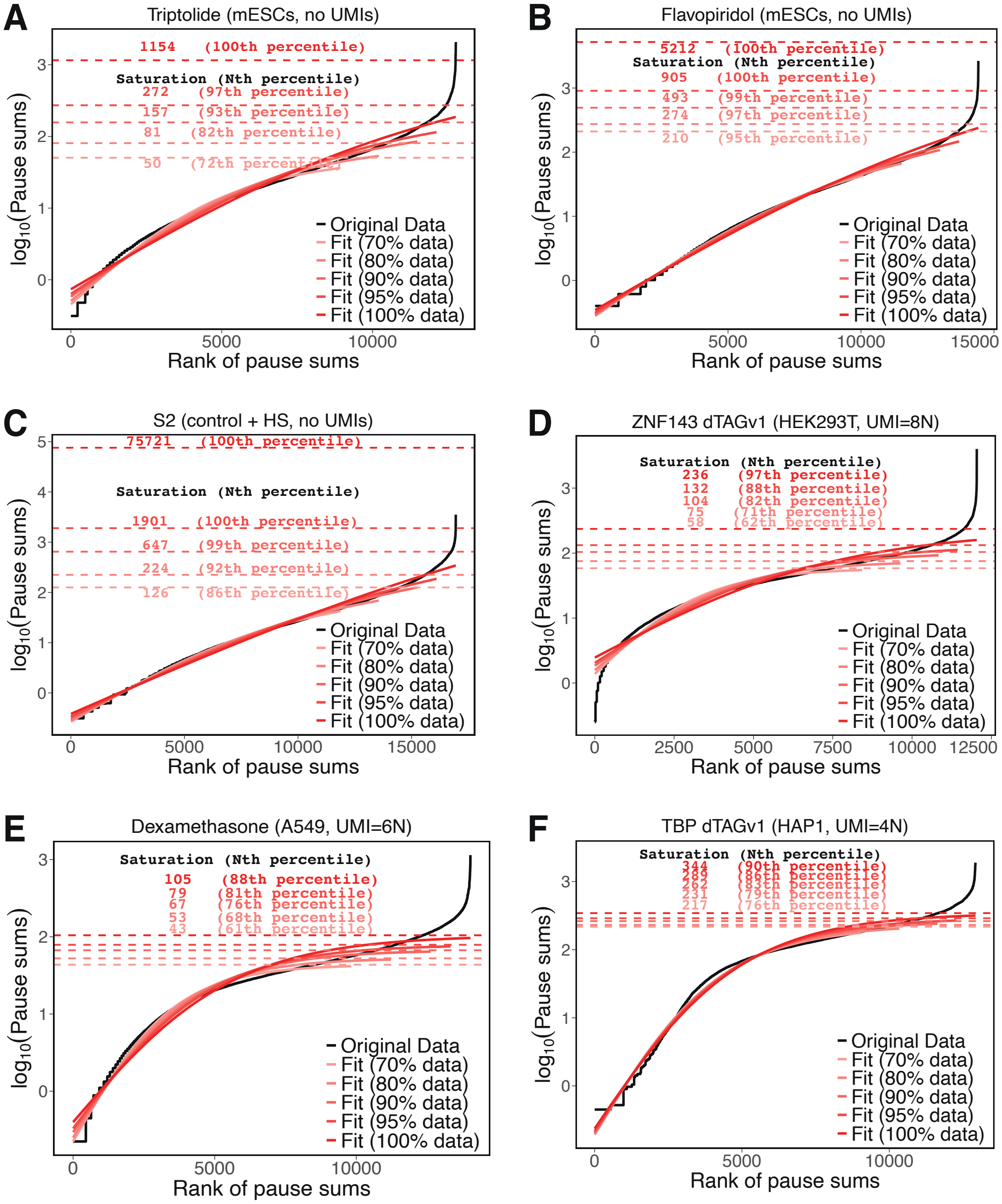

Fig. S1. Saturation curves for datasets with Unique Molecular Identifiers (UMIs) provide estimates of RNA polymerase occupancy. The plots show the saturation curves and values from fits to the hyperbolic tangent function for datasets lacking Unique Molecular Identifiers (UMIs) (A-C) or containing UMIs (D-F). Datasets lacking UMIs do not have stable saturation values when ranges of pause sum percentiles are used to fit the saturation curve.

bioRxiv preprint doi: https://doi.org/10.1101/2025.03.27.645809; this version posted March 28, 2025. The copyright holder for this preprint (which was not certified by peer review) is the author/funder, who has granted bioRxiv a license to display the preprint in perpetuity. It is made available under aCC-BY 4.0 International license.

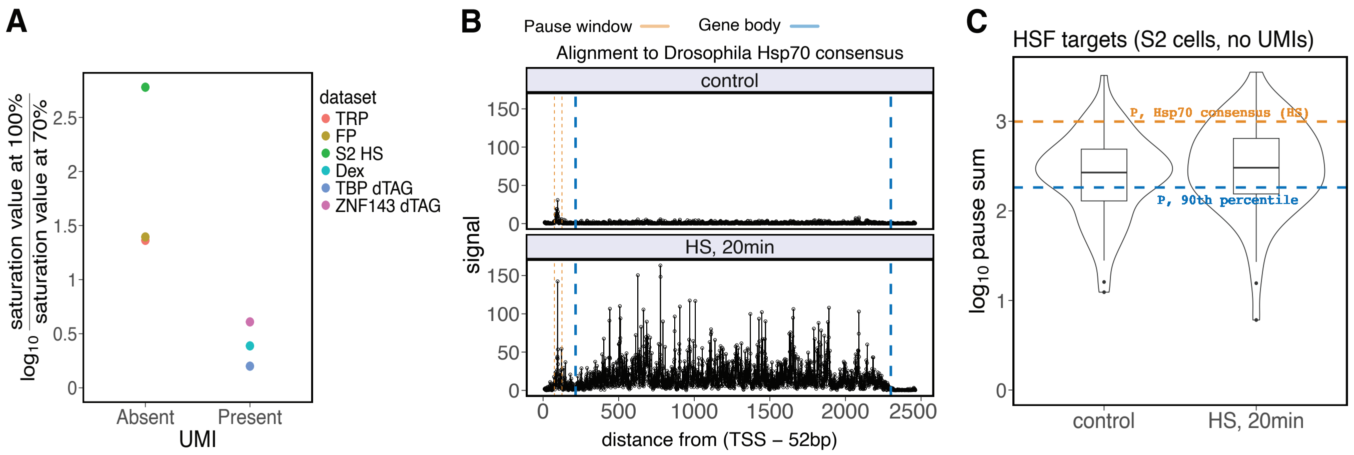

Fig. S2. Saturation values from datasets lacking Unique Molecular Identifiers (UMIs) are unreliable as maximum occupancy signal of RNA polymerase. A) The ratio of saturation values derived from fits to 100% of the data compared to 70% of the data (ignoring the top 30% of pause sum genes) is substantially higher for datasets without UMIs, ranging from 23-fold to 600-fold. In contrast, datasets with UMIs show much lower variability, with ratios ranging from 1.6-fold to 4-fold. B) These plots show the normalized read signal for the *Hsp70* consensus sequence from the dataset (33) used in this study. C) The pause sum of *Hsp70* (P = 992) is five times higher than the pause sum of the gene at the 90th percentile (P = 182). The violin plot represents the pause sums for all HSF-activated genes, most of which are higher than the 90th percentile value. Considering that *Hsp70* has a fully occupied pause region during heat shock (85), the assumption that the 90th percentile pause sum represents the maximum occupancy signal is not supported in this dataset lacking UMIs.

bioRxiv preprint doi: https://doi.org/10.1101/2025.03.27.645809; this version posted March 28, 2025. The copyright holder for this preprint (which was not certified by peer review) is the author/funder, who has granted bioRxiv a license to display the preprint in perpetuity. It is made available under aCC-BY 4.0 International license.

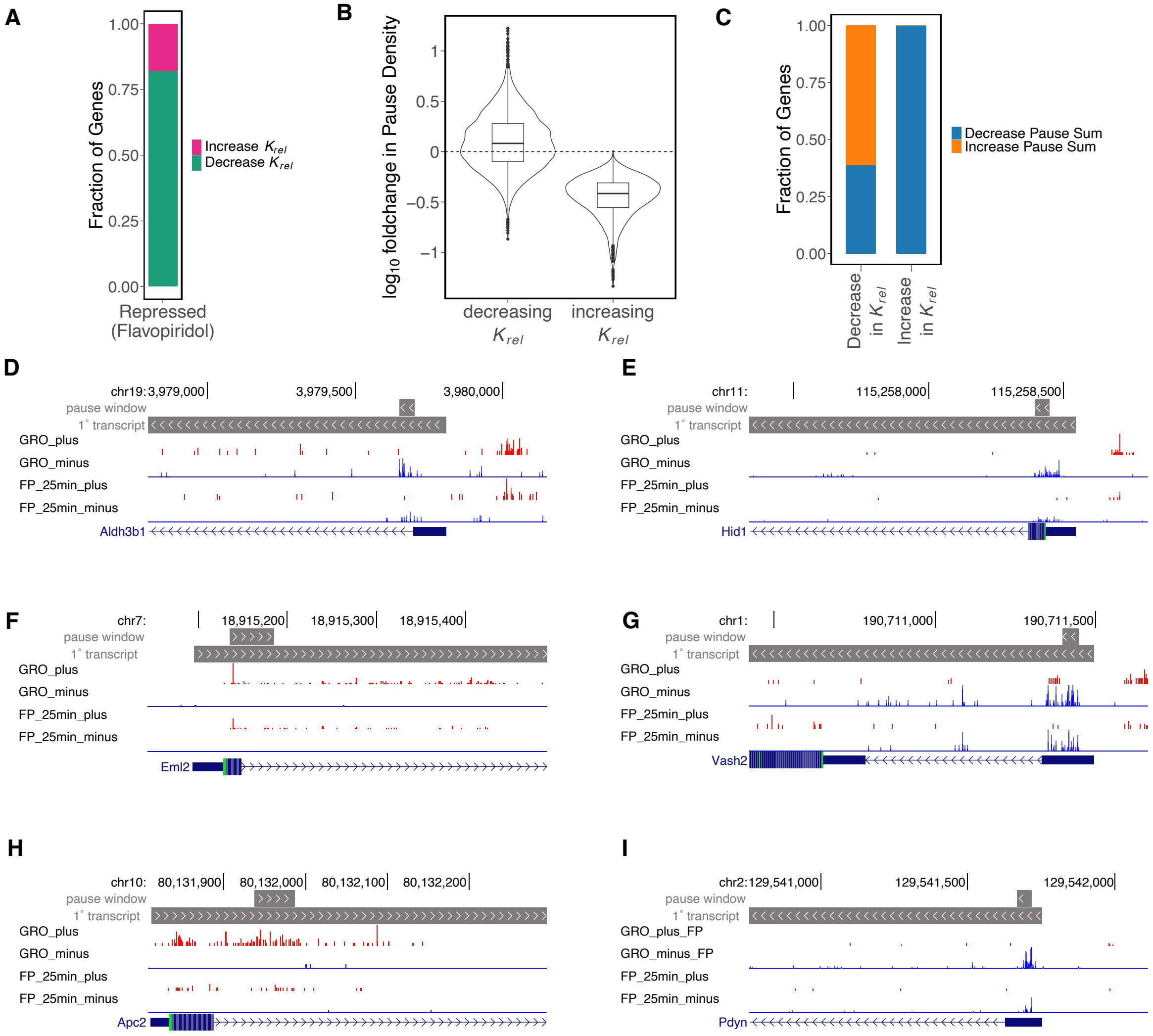

Fig. S3. Genes with increased pause release after flavopiridol treatment have atypical read distributions. A) Approximately 82% of flavopiridol-repressed genes decrease their pause release rate (k_{rel}). B) All genes with increased pause release rate also have a decrease in pause density. C) Sixty-one percent of repressed genes with decreased pause release have an expected increase in pause density after flavopiridol treatment. D-I) The UCSC Genome Browser snapshots show representative genes with increased pause release (k_{rel}) and decreased pause density. High pause signals under untreated conditions in these genes may result from the lack of UMIs in the libraries (11).

bioRxiv preprint doi: https://doi.org/10.1101/2025.03.27.645809; this version posted March 28, 2025. The copyright holder for this preprint (which was not certified by peer review) is the author/funder, who has granted bioRxiv a license to display the preprint in perpetuity. It is made available under aCC-BY 4.0 International license.

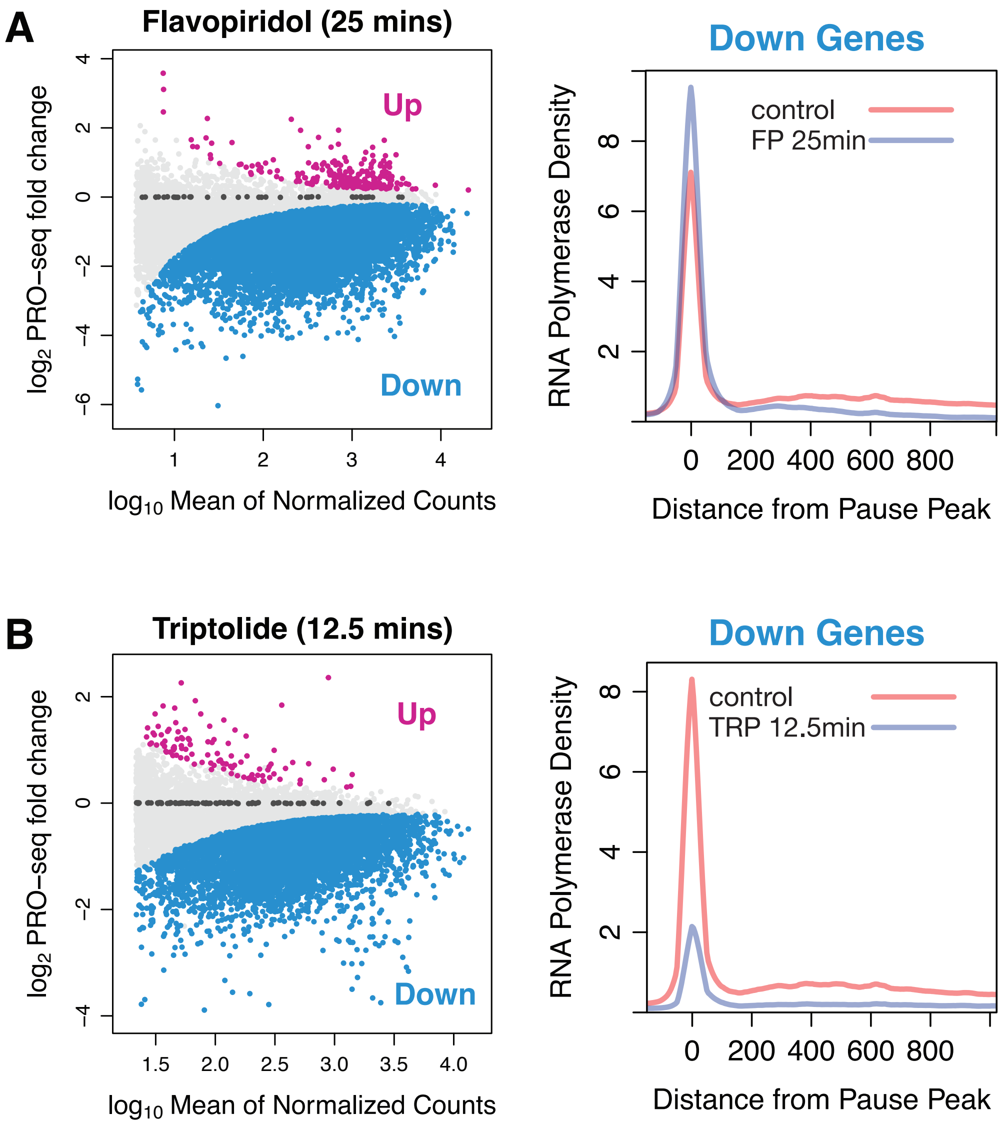

Fig. S4. Flavopiridol and Triptolide induce genome-wide repression and changes in RNA polymerase distribution. A) Flavopiridol (FP) treatment for 25 mins (11) resulted in 12994 *Down* and 310 *Up* genes. Composite profiles of the *Down* genes show an increase in RNA polymerase density in pause window upon FP treatment. B) Triptolide treatment for 12.5 mins (11) resulted in 9632 *Down* and 104 *Up* genes. The *Down* genes have a decrease in RNA polymerase density in pause window and gene body upon triptolide treatment.

bioRxiv preprint doi: https://doi.org/10.1101/2025.03.27.645809; this version posted March 28, 2025. The copyright holder for this preprint (which was not certified by peer review) is the author/funder, who has granted bioRxiv a license to display the preprint in perpetuity. It is made available under aCC-BY 4.0 International license.

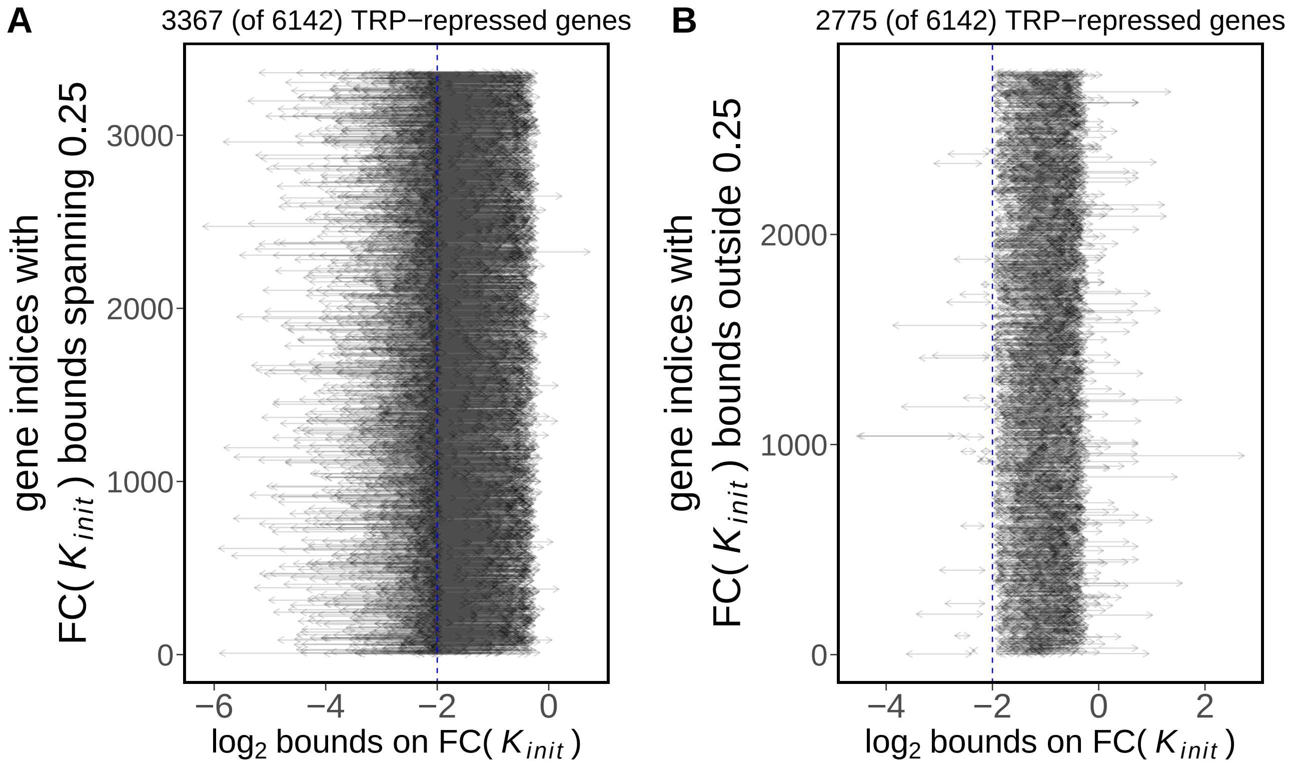

Fig. S5. The model restrains the fold change initiation rates after triptolide inhibition for each genes within a calculated range. We consider repressed genes from a dataset that used 0.5 µM triptolide for 12.5 mins (11). A previous study reported a 0.25 fold change (dashed vertical line) in K_{init} upon treatment with 1µM triptolide at approximately 12 mins (61). We found 3367 (55% of 6142) TRP-repressed genes had bounds on changes in initiation rate spanning 0.25 (A). 2775 (45% of 6142) TRP-repressed genes had bounds on changes in initiation rate not spanning 0.25 (B).

bioRxiv preprint doi: https://doi.org/10.1101/2025.03.27.645809; this version posted March 28, 2025. The copyright holder for this preprint (which was not certified by peer review) is the author/funder, who has granted bioRxiv a license to display the preprint in perpetuity. It is made available under aCC-BY 4.0 International license.

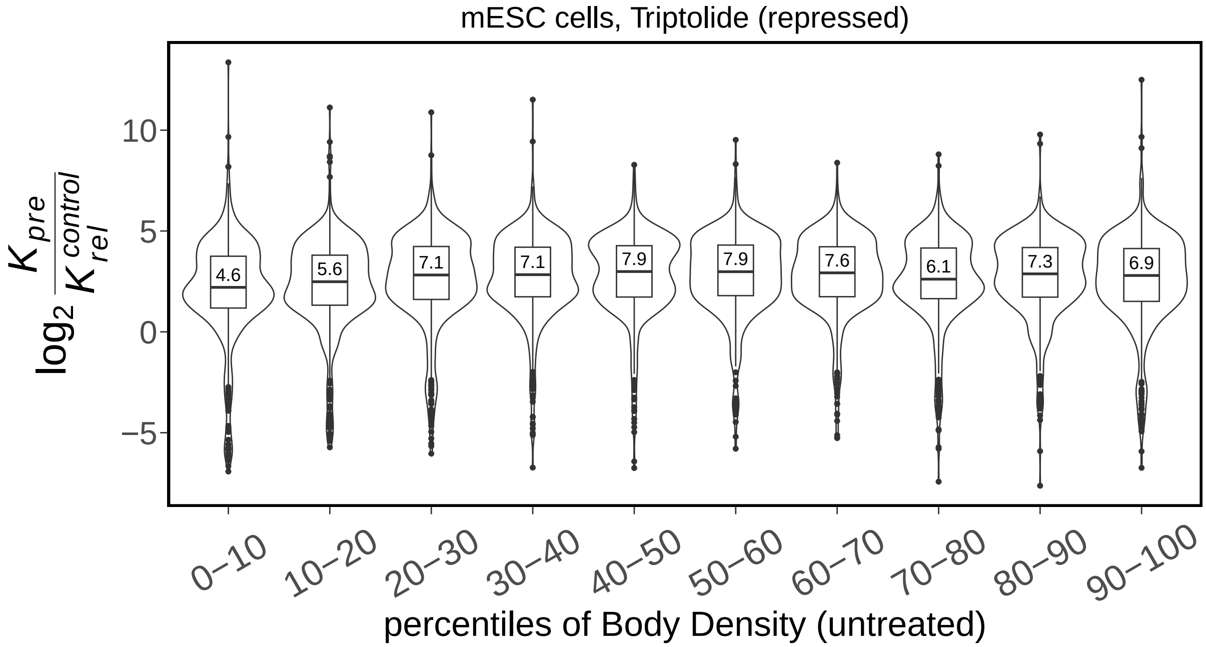

Fig. S6. Premature termination is faster than pause release regardless of gene expression levels. We assumed that the repressed genes decrease initiation by ~75% and calculated ratio of premature termination and pause release rates for the indicated gene expression quantiles.

bioRxiv preprint doi: https://doi.org/10.1101/2025.03.27.645809; this version posted March 28, 2025. The copyright holder for this preprint (which was not certified by peer review) is the author/funder, who has granted bioRxiv a license to display the preprint in perpetuity. It is made available under aCC-BY 4.0 International license.

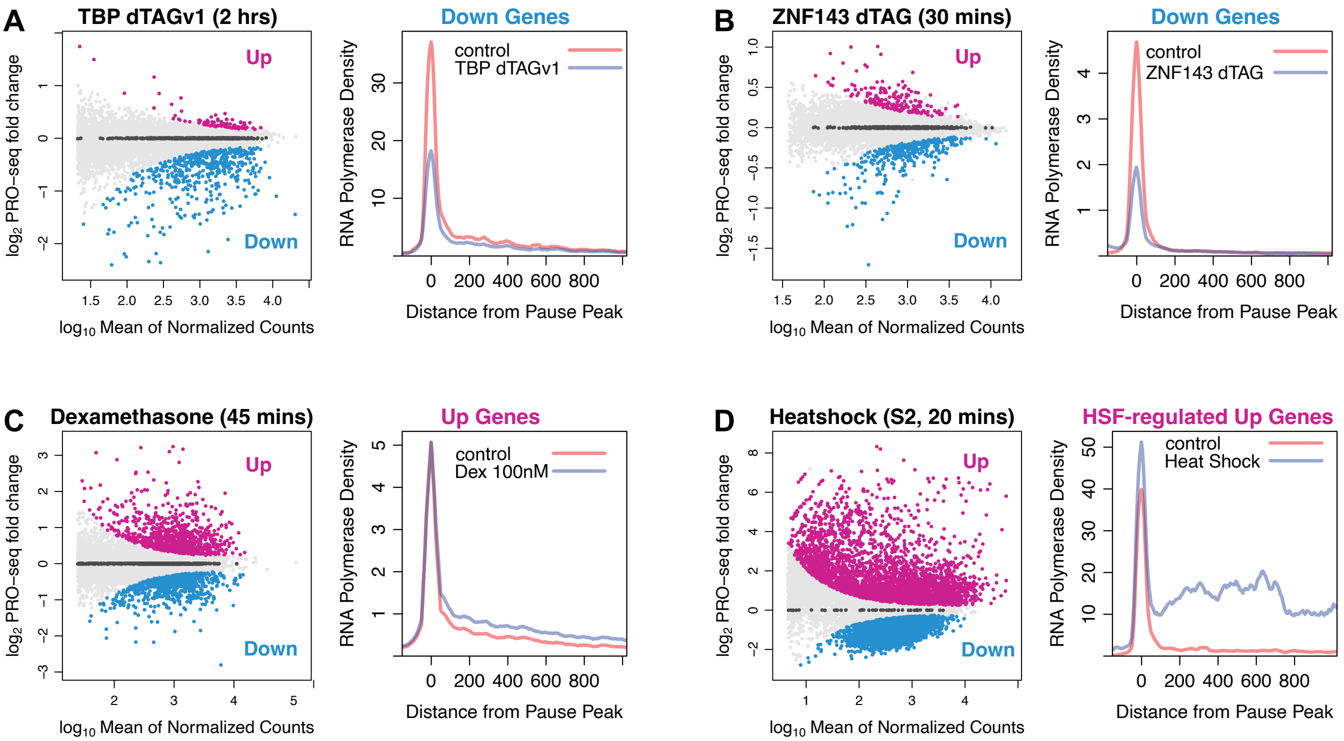

Fig. S7. Acute treatments induce genomic changes in gene expression and RNA polymerase distribution. A) Degradation of TBP (43) led to 493 *Down* and 86 *Up* genes. The *Down* genes have reduced RNA polymerase density in pause region upon TBP degradation. B) Rapid degradation of ZNF143 (44) led to 358 *Down* genes and 181 *Up* genes. The *Down* genes decrease pause density. C) Dexamethasone treatment for 45 mins (45) resulted in 862 *Up* and 738 *Down* genes. The *Up* genes have increased RNA polymerase density in pause window and gene body upon dexamethasone treatment. D) Heat shock (HS) in *Drosophila S2* cells resulted in 3922 *Up* genes, although many of these genes are false positives due to read-through transcription (33). The genes that are confidently HSF-activated (Materials & Methods) increase RNA polymerase density in both the pause region and gene body.

bioRxiv preprint doi: https://doi.org/10.1101/2025.03.27.645809; this version posted March 28, 2025. The copyright holder for this preprint (which was not certified by peer review) is the author/funder, who has granted bioRxiv a license to display the preprint in perpetuity. It is made available under aCC-BY 4.0 International license.

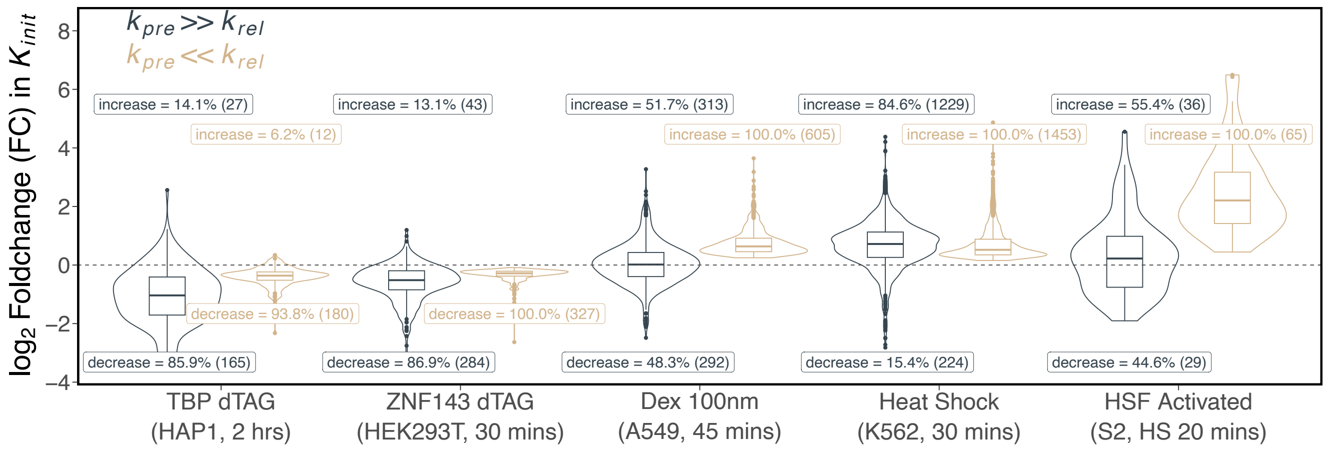

Fig. S8. Changes in initiation rate for datasets used in this study vary substantially depending on the $k_{rel} >> k_{pre}$ assumption. This figure reproduces the data from 4 and includes changes in initiation rates for faster pause release as well ($k_{rel} >> k_{pre}$).

bioRxiv preprint doi: https://doi.org/10.1101/2025.03.27.645809; this version posted March 28, 2025. The copyright holder for this preprint (which was not certified by peer review) is the author/funder, who has granted bioRxiv a license to display the preprint in perpetuity. It is made available under aCC-BY 4.0 International license.

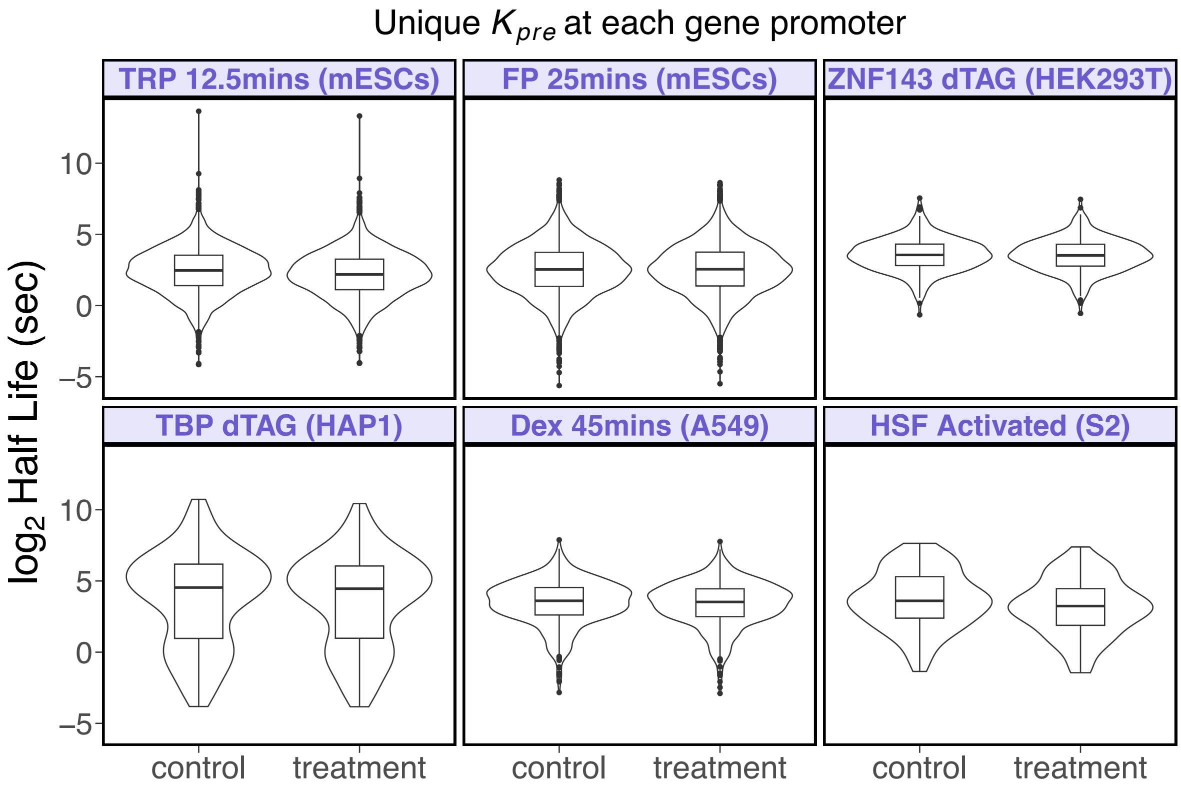

Fig. S9. Turnover times of paused polymerases can become extremely rapid, reaching sub-second timescales at high premature termination rates. Violin plots illustrate the distribution of paused RNA polymerase half lives if premature termination rates at each gene promoter are ten times faster than pause release rates (i.e., $k_{pre} = 10 \times k_{rel}$). The median half-life of paused RNA polymerases across all datasets and treatments is 5.83 seconds, with an inter-decile range of 1.2 to 26 seconds.

bioRxiv preprint doi: https://doi.org/10.1101/2025.03.27.645809; this version posted March 28, 2025. The copyright holder for this preprint (which was not certified by peer review) is the author/funder, who has granted bioRxiv a license to display the preprint in perpetuity. It is made available under aCC-BY 4.0 International license.

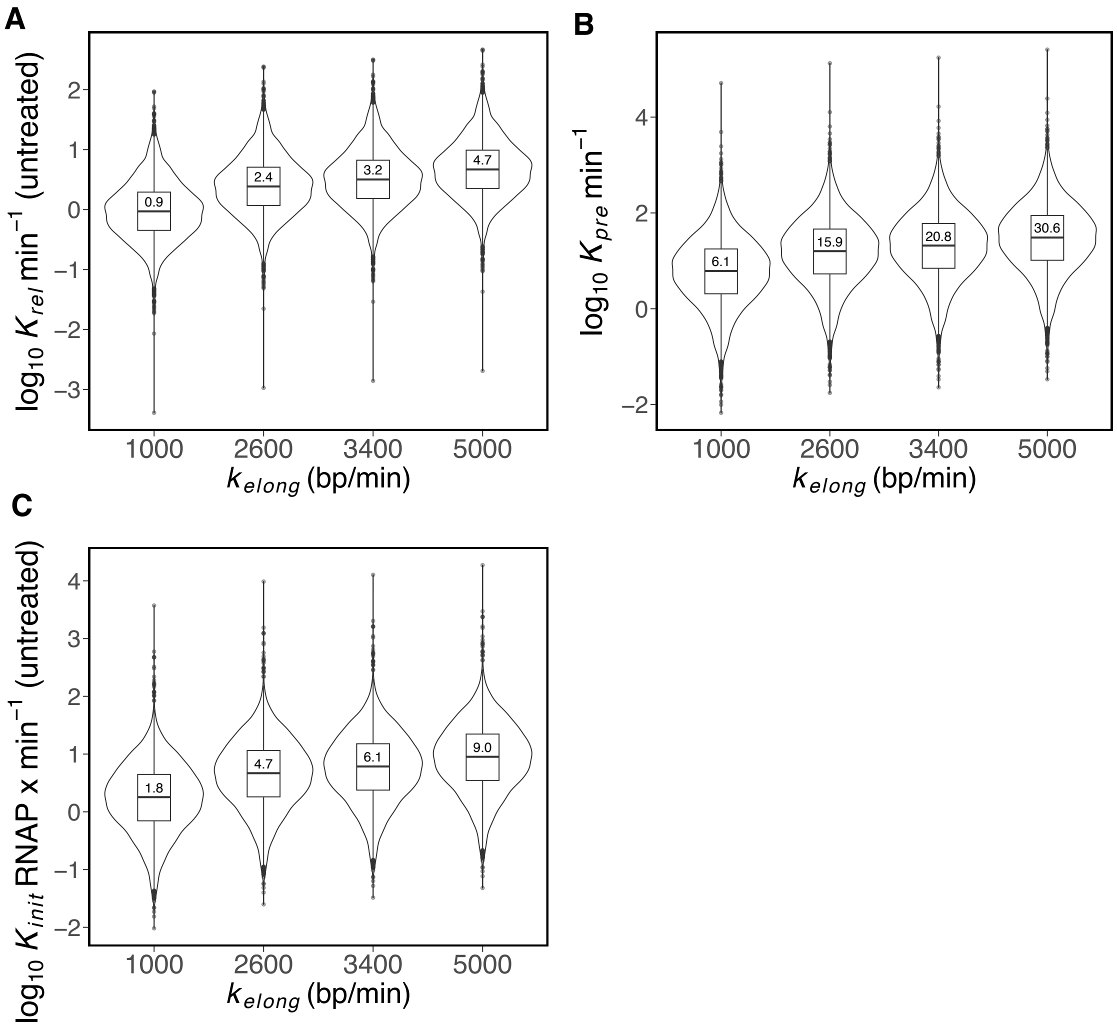

Fig. S10. The rates of pause release, premature termination, and initiation are proportional to elongation rates. A) We varied elongation rates from 1–5 kb/min. Under untreated conditions for repressed genes under TRP inhibition (11), pause release rates (k_{rel}) had interdecile ranges of 0.24–4.1 events/min, 0.6–10.8 events/min, 0.82–14.1 events/min, and 1.2–20.7 events/min for elongation rates of 1, 2.6, 3.4, and 5 kb/min, respectively. Median values are indicated in the boxplots. B) We calculated premature termination rates (k_{pre}) based on pause release using Eq. 14. The fold change in initiation rate (k_{init}) was set to 0.25. The inter-decile ranges of premature termination rates were 0.7-43.5 events/min, 1.8-113.1 events/min, 2.3-148.0 events/min and 3.4-217.5 events/min for elongation rates of 1, 2.6, 3.4, and 5 kb/min, respectively. Median values are indicated. C) We derived initiation rates (k_{init}) using values of premature termination and elongation rates (Eq. 12). The inter-decile ranges of initiation rates were 0.3-9.5 RNAP/min, 0.72-24.8 RNAP/min, 0.94-32.4 RNAP/min and 1.4-47.7 RNAP/min for 1, 2.6, 3.4, and 5 kb/min, respectively. Median values are indicated.

bioRxiv preprint doi: https://doi.org/10.1101/2025.03.27.645809; this version posted March 28, 2025. The copyright holder for this preprint (which was not certified by peer review) is the author/funder, who has granted bioRxiv a license to display the preprint in perpetuity. It is made available under aCC-BY 4.0 International license.

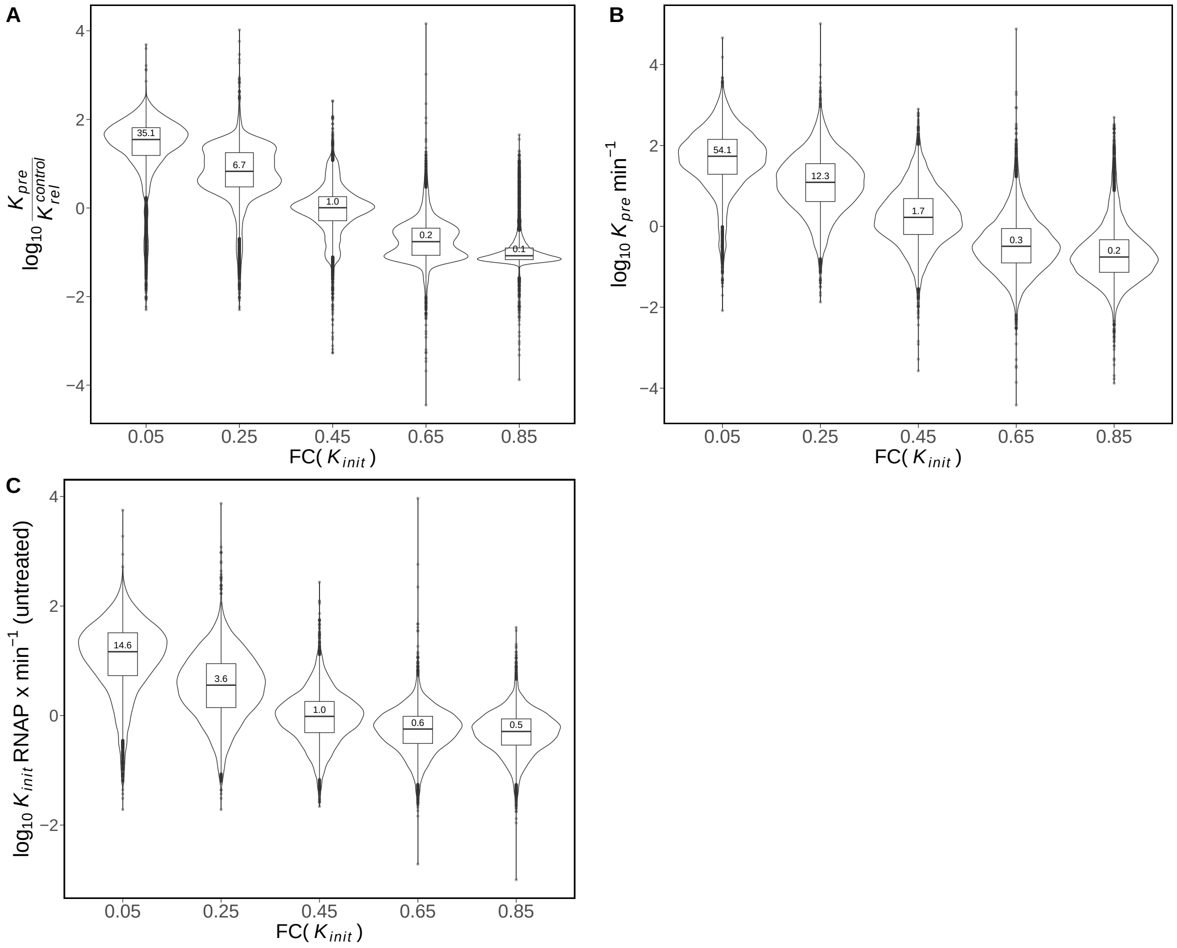

Fig. S11. Parameter sensitivity analysis indicates that the premature termination to pause release rate ratio is >1 if triptolide reduces initiation rate to less than 0.4 the control initiation rate. A) We varied the global decrease in k_{init} from 95% to 15%, corresponding to a fold change (FC) in k_{init} from 0.05 to 0.85 for repressed genes under TRP-inhibition (11). We set the elongation rate at a representative value of 2000 bp/min. We then calculated ratio of premature termination to pause release using Eq. 14. Median ratios are indicated. The inter-decile range at FC (k_{init}) = 0.05 is 4.0 to 108, decreasing to inter-decile range of 0.06-0.6 at FC (k_{init}) = 0.65 and 0.06-0.3 at FC (k_{init}) = 0.85. B) We estimated premature termination (k_{pre}) based on the ratio in (A). Considering elongation rate of 2000 bp/min, the pause release rate (Eq. 11) has a median of 3.9 events/min with an inter-decile range of 0.5-8.3 events/min. The inter-decile range of k_{pre} is 5.5-338 events/min at FC (k_{init}) = 0.05, decreasing to 0.05 - 2.7 events/min at FC (k_{init}) = 0.65 and 0.04-1.7 events/min at FC (k_{init}) = 0.85. C) We calculated initiation rate as indicated in Eq. 12. The inter-decile range changes from 1.5-62 RNAP/min at FC (k_{init}) = 0.05 which change to 0.16 - 1.5 RNAP/min at FC (k_{init}) = 0.65 and 0.15-1.4 RNAP/min at FC (k_{init}) = 0.85.

bioRxiv preprint doi: https://doi.org/10.1101/2025.03.27.645809; this version posted March 28, 2025. The copyright holder for this preprint (which was not certified by peer review) is the author/funder, who has granted bioRxiv a license to display the preprint in perpetuity. It is made available under aCC-BY 4.0 International license.

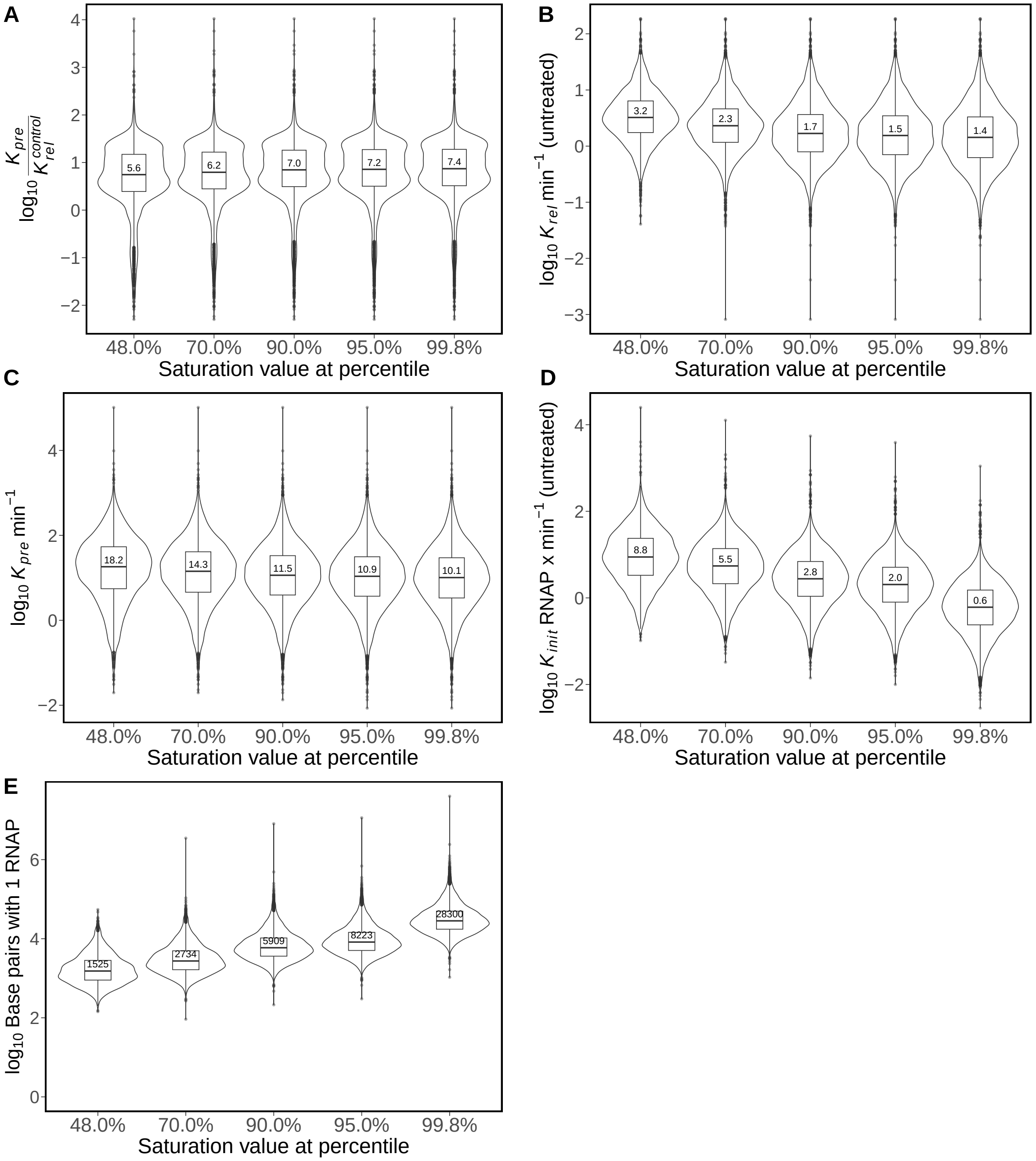

Fig. S12. Increasing signal from a fully occupied pause region decreases rates of pause release, initiation and premature termination. We selected saturation values at different percentiles of ranked pause sums for repressed genes under TRP-inhibition (11). To achieve 25-fold dynamic range around the saturation value considered at 90th percentile of ranked pause sums (P = 169, 90th percentile), we varied saturation values from 34 (at 48th percentile) to 844 (at 99.8th percentile). A) We calculated ratios of premature termination and pause release using Eq. 14. We set the foldchange (FC) in initiation rate to 0.25 and elongation rate to 2000 bp/min. The inter-decile ranges of ratio increases from 0.7-27.0 at 48th percentile to 1.44-31.5 at 99.8th percentile. B) The pause release rates (Eq. 11) have an inter-decile range of 0.9-11.6 events/min at 48th percentile to 0.3-7.24 events/min at 99.8th percentile. Median values are indicated on the boxplots. C) The premature termination rates (Eq. 14) have an inter-decile range of 1.4-133.9 events/min at 48th percentile to 1.2-74.7 events/min at 99.8th percentile. D) The initiation rates (Eq. 12) have an inter-decile range of 1.3-52.0 polymerase/min at 48th percentile and 0.1-3.2 polymerase/min at 99.8th percentile. E) We calculated the density of RNA Polymerase on gene body using Eq. 13. The RNA polymerases were less densely spaced on gene body with increasing signal of a fully occupied pause region. The inter-decile ranges were 1 polymerase every 585 bp - 5.4 kb at 48th percentile which decreased to 1 polymerase every 2.4 kb - 19.7 kb at 90th percentile and to 1 polymerase every 11.6 kb - 93.2 kb at 99.8th percentile.

bioRxiv preprint doi: https://doi.org/10.1101/2025.03.27.645809; this version posted March 28, 2025. The copyright holder for this preprint (which was not certified by peer review) is the author/funder, who has granted bioRxiv a license to display the preprint in perpetuity. It is made available under aCC-BY 4.0 International license.

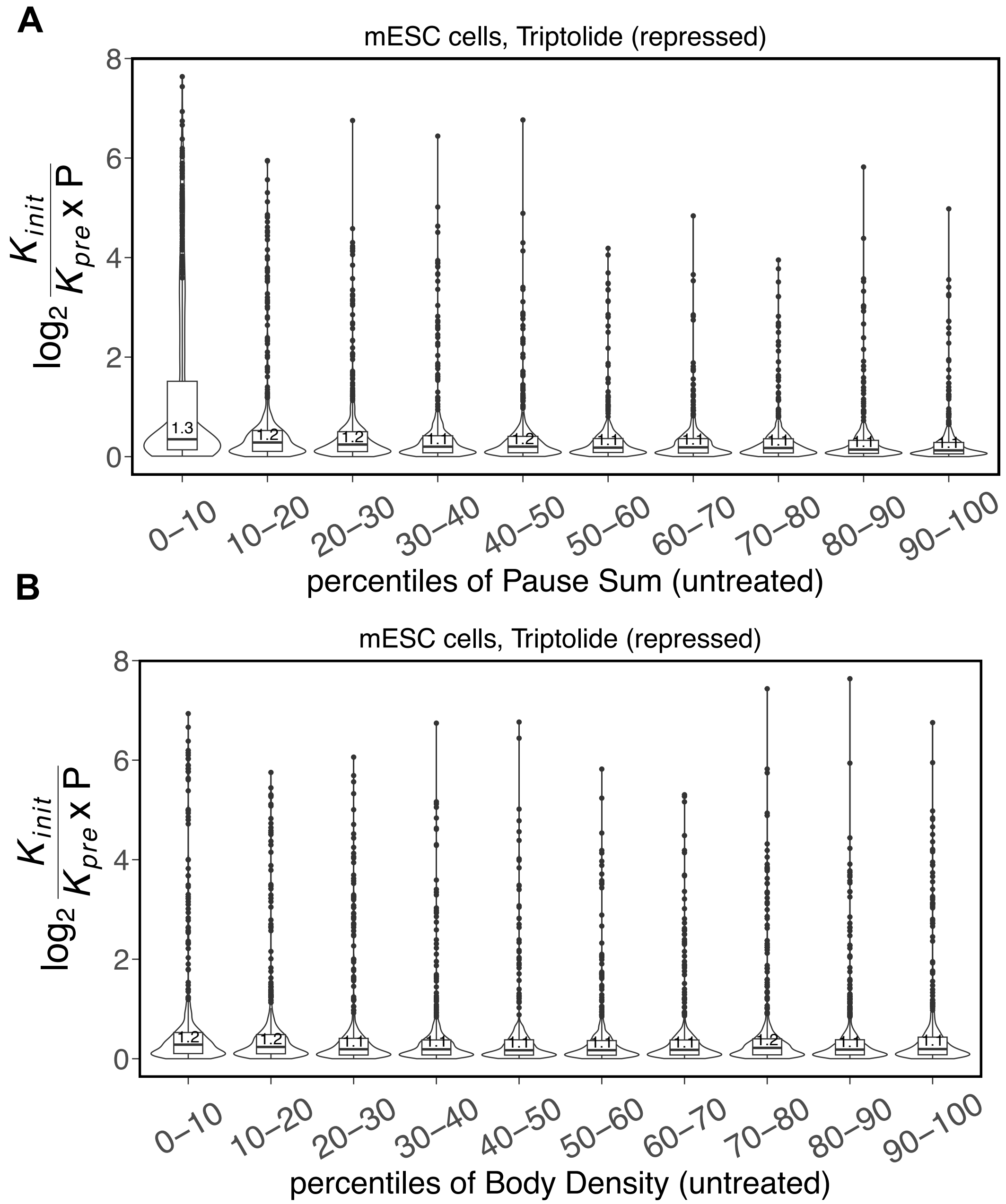

Fig. S13. Most initiated RNA polymerases prematurely terminate. We categorized genes based on percentiles of paused polymerase density (A) and gene expression (B). We then estimated the ratio of initiation rate to effective premature termination ($k_{pre} \times P$) under untreated conditions. The median values ranged from 1.1 to 1.2, with a highly skewed distribution centered around 1. This suggests that the effective premature termination rate ($k_{pre} \times P$) is generally equivalent to k_{init}, regardless of paused polymerase density or gene expression levels.

bioRxiv preprint doi: https://doi.org/10.1101/2025.03.27.645809; this version posted March 28, 2025. The copyright holder for this preprint (which was not certified by peer review) is the author/funder, who has granted bioRxiv a license to display the preprint in perpetuity. It is made available under aCC-BY 4.0 International license.

Table S1

Treatment	Cell line	Species	Time	Citation	GEO Identifier
Triptolide	v6.5	Mouse	12.5 mins	Jonkers et al., 2014	GSE48895
Flavopiridol	v6.5	Mouse	25 mins	Jonkers et al., 2014	GSE48895
Dexamethasone	A549	Human	45 mins	Wissink et al., 2021	GSE168767
ZNF143 dTAG	HEK293T	Human	30 mins	Dong et al., 2024	GSE266491
TBP dTAG	HAP1	Human	2 hrs	Santana et al., 2022	GSE194153
Heat Shock	S2	Drosophila	20 mins	Duarte et al., 2016	GSE77607

Table S1. Gene Expression Omnibus (GEO) accession numbers for datasets used in this study.

bioRxiv preprint doi: https://doi.org/10.1101/2025.03.27.645809; this version posted March 28, 2025. The copyright holder for this preprint (which was not certified by peer review) is the author/funder, who has granted bioRxiv a license to display the preprint in perpetuity. It is made available under aCC-BY 4.0 International license.

Table S2

Study	Premature Termination	Pause Release
Zimmer et al., 2021	median 0.11 min^{-1}; inner 90% range 0.027–0.23 min^{-1}	median 0.027 min^{-1}; inner 90% range 0.0015–0.31 min^{-1}
Steurer et al., 2018	Frequency: 12 out of 13 Pol2 terminate at promoter proximal pause region (~92.4%, Fig. 3B)	Frequency: 1 out of 13 Pol2 proceed into elongation (7.6%, Fig. 3B)

Table S2. Multiple independent studies report faster premature termination rate compared to pause release.
